# Various Structural Types of Cyanide-Bridged Fe^III^–Mn^III^ Bimetallic Coordination Polymers (CPs) and Polynuclear Clusters Based-on A New *mer-*Tricyanoiron(III)Building Block: Synthesis, Crystal Structures, and Magnetic Properties

**DOI:** 10.3390/polym11101585

**Published:** 2019-09-27

**Authors:** Wenlong Lan, Xiaoyun Hao, Yong Dou, Zhen Zhou, Lu Yang, Hui Liu, Dacheng Li, Yunhui Dong, Lingqian Kong, Daopeng Zhang

**Affiliations:** 1 College of Chemical and Chemical Engineering, Shandong University of Technology, Zibo 255049, China; 17090419074@163.com (W.L.); 17865917891@163.com (X.H.); dou18753363463@163.com (Y.D.); zhouzhen@sdut.edu.cn (Z.Z.); Yanglu@sdut.edu.cn (L.Y.); liuhui1030@163.com (H.L.); dyh651118@163.com (Y.D.); 2 College of Chemical and Chemical Engineering, Liaocheng University, Liaocheng 252059, China; lidacheng62@163.com; 3 Dongchang College of Liaocheng University, Liaocheng 252059, China; lingqiankong@126.com

**Keywords:** coordination polymer, cyanide-bridged, new building block, single crystal structure, magnetism

## Abstract

Four cyanide-bridged Fe^III^–Mn^III^ complexes {[Fe(qxcq)(CN)_3_][Mn(L^1^)(H_2_O)]}[Mn(L^1^)(H_2_O)(CH_3_OH)](ClO_4_)·1.5MeOH·0.5H_2_O (L^1^ = *N,N′-bis*(3-methoxy-5-bromosalicylideneiminate) (2), {[Fe(qxcq)(CN)_3_][Mn(L^2^)]}_2_·0.5H_2_O (L^2^ = *N,N′*-ethylene-*bis*(3-ethoxysalicylideneiminate)) (3), [Fe(qxcq)(CN)_3_][Mn(L^3^)] (L^3^ = *bis*(acetylacetonato)ethylenediimine) (4), [Fe(qxcq)(CN)_3_][Mn(L^4^)]·1.5MeOH·0.5CH_3_CN·0.25H_2_O (L^4^ = *N,N′*-(1,1,2,2-tetramethylethylene)bis(salicylideneiminate)) (5), were prepared by assembling a new structurally characterized *mer*-tricyanoiron(III) molecular precursor (Ph_4_P)[Fe(qxcq)(CN)_3_]·0.5H_2_O (qxcq^−^ = 8-(2-quinoxaline-2-carboxamido)quinoline anion) (1) and the corresponding manganese(III) Schiff base compound. Complexes 2and 3containa cyanide-bridged heterobimetallic dinuclear entity, which can be further dimerized by self-complementary H-bond interactions through the coordinated water molecule from one complex and the free O_4_unit from the adjacent complex. Complexes 4 and 5 area one-dimensional coordination polymer (CP) comprised of the repeated [Mn(Schiffbase)-Fe(qxcq)(CN)_3_] units. Complex 4 shows a linear-chain conformation with two *trans*-located cyano groups bridgingthe neighboring Mn units, while complex 5 is a zigzag-like 1D CP, where the two cyano groups in *cis* configurationfunction as bridges. In bothcomplexes 4 and 5, the inter-chain π–πstack interactions within the aromaticrings of cyanide precursor extend the 1D chain into the supermolecular 2D networks. The magnetic property has been experimentally studied and theoretically fitted over the four Fe(III)-Mn(III) complexes, revealing the antiferromagnetic interaction in complexes 2 and 4 and the unusual ferromagnetic coupling in complexes 3 and 5 between the Fe(III) ion and the Mn(III) ion bridged by the cyano group. Furthermore, the different magnetic coupling nature has been analyzed on the basis of the magneto-structure correlation of the *mer*-tricyanometallate-based Fe(III)-Mn(III) magnetic system.

## 1. Introduction

The research on molecule-based magnetic materials has always been within the hottest topics of interests for chemical and physical researchersdue to their potential values in molecule devices, information memorizer, and quanta computers, et. al. [1,2,3,4]. Among which, since their magnetic coupling nature between neighboring paramagnetic metal centers through the cyanogroup and the molecular topological structures can be relatively easily predicted and controlled, the cyanide-bridged magnetic systems have been widely and intensely studied. Recently, with the aim not only of further disclosing the magneto-structure correlation but also more importantly for the great potential in many high-tech fields, a large numberof molecular magnetic materials containing a cyanide linkagewith different structural types have been synthesized and magnetically studied.Many of the reported cyanide-bridged magnetic assemblies showed very interesting magnetic properties [5,6,7,8,9,10,11,12,13,14] including single-molecule magnet (SMM) and single-chain magnet (SCM), which behave as nanosized magnets under the blocking temperature because of the energy barrierscoming from the large ground spin state (ST) and the strong easy axial anisotropy (D) [15,16,17].

**Scheme 1:**
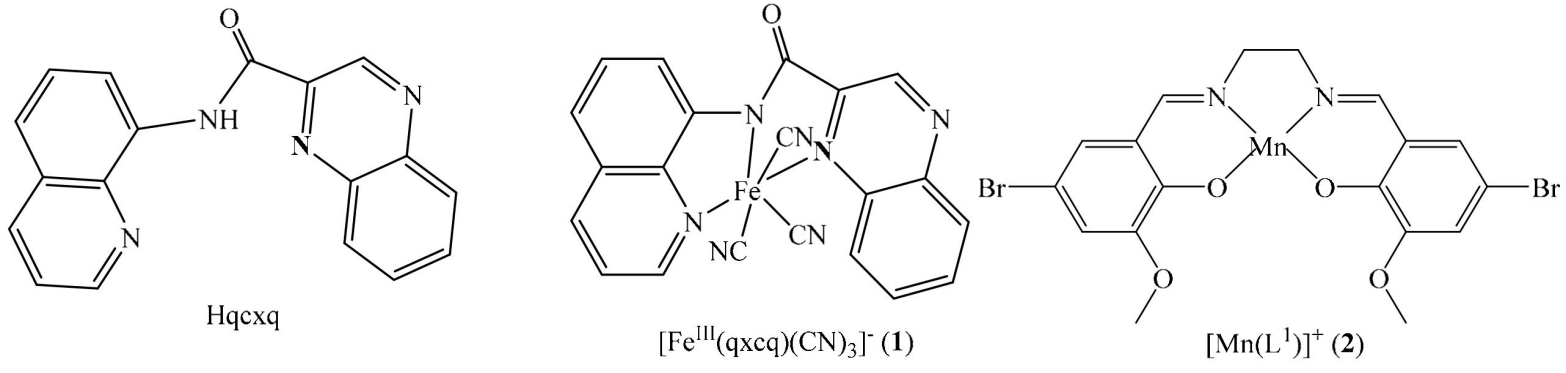

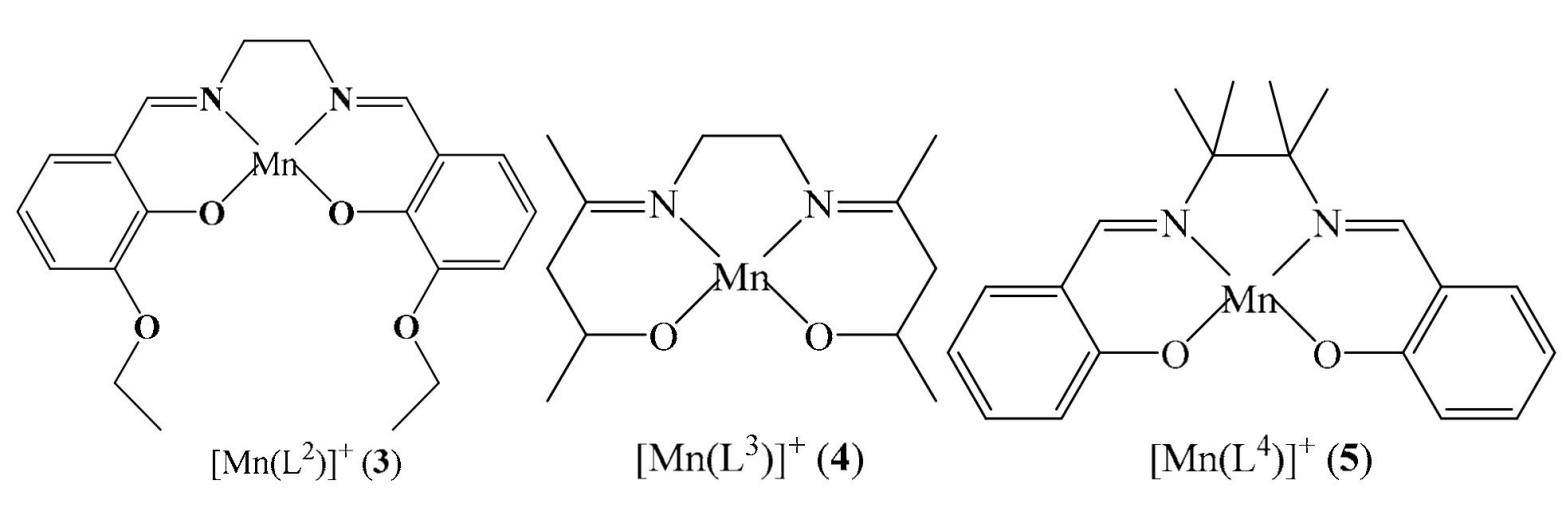
The organic ligand, *mer*-[Fe^III^(qxcq)(CN)_3_]^−^ anion and the Mn^III^(Schiffbase) employed in this paper.

Previous reports have proven that modified polycyanometallates [M(L)_m_(CN)_n_]^z−^(M = Fe, Cr, Mo, W; L = mono- or multi-dentate blocking ligand) are good candidates for assembling lowdimensional molecule-based magnetism complexes [5,6,13,14,18,19]. The introduction of organic ligand(s) into the cyanometallate is very helpful for tuning the assemble architectures and molecular topologies of the resulting complexes. More than that, the blocking ligand(s) can define the magnetic orbitals used to describe the single electrons in paramagnetic metal ions and therefore affect the magnetic interaction mechanism, thereforeofferinga valuable chance for researchers to regulate and control the magnetic property of the complex system. Up to now, many 0D discrete clusters and 1D infinite coordination polymers have been successfully obtained by assembling the decorated polycyanometallates such as the blocked *trans*-dicyano-, *fac*-tricyano-, *mer*-tricyano- and tetracyanometallate, and the paramagnetic compound with or without the auxiliary ligand [18,19,20,21,22,23]. Several reports from our and other groups on the *mer*-[Fe(L](CN)_3_]^-/2-^-based cyanide-bridged complexes, where L represents the equatorial in-plane ligands with relative large steric effect for efficiently lowering the dimensionality of the target complex such as *bis*(2-pyridylcarbonyl)amidate (bpca) [24,25], 8-(pyridine-2-carboxamido)quinoline (pcq) [26,27,28], 8-(pyrazine-2-carboxamido)quinoline (pzcq) [29], 8-(5-methylpyrazine-2-carboxamido)quinoline (mpzcq) [30], 8-(2-quinoline-2-carboxamido)quinoline (qcq) [31],*N*-(quinolin-8-yl)isoquinoline-1-carboxamide anion (iqc) [32], and *bis*(2-benzimidazolyl)pyridine (bbp) [33,34] showed that the *mer*-[Fe(L](CN)_3_]^-/2-^ type precursors are excellent candidates for constructing interesting magnetic assemblies. Here, a new structurally characterized *mer*-tricyanoiron(III) building block **1** (Scheme 1) was first employed to assemble with the manganese Schiff base compounds (Scheme 1), leading to a series of new CN-bridged Fe(III)-Mn(III) complexes with various structural types formulated as {[Fe(qxcq)(CN)_3_][Mn(L^1^)(H_2_O)]}[Mn(L^1^)(H_2_O)(CH_3_OH)](ClO_4_)·1.5MeOH·0.5H_2_O (L^1^ = *N,N′-bis*(3-methoxy-5-bromosalicylideneiminate) (2), {[Fe(qxcq)(CN)_3_][Mn(L^2^)]}_2_·0.5H_2_O (L^2^ = *N,N′*-ethylene-*bis*(3-ethoxysalicylideneiminate)) (3), [Fe(qxcq)(CN)_3_][Mn(L^3^)] (L^3^ = *bis*(acetylacetonato)ethylenediimine) (4), and [Fe(qxcq)(CN)_3_][Mn(L^4^)]·1.5MeOH·0.5CH_3_CN·0.25H_2_O (L^4^ = *N,N′*-(1,1,2,2-tetramethylethylene)bis(salicylideneiminate)) (**5**).

## 2. Experimental

### 2.1. General Procedures and Materials

The preparation reaction for all compounds was finished at room temperaturein air, and all of the solvents and chemicals used were reagent grade without any further purification. The *mer*-[Fe^III^(qxcq)(CN)_3_]^−^ precursor was preparedby referencing the method used to prepared other *mer*-tricyanoiron(III) compounds [35]. The Schiff base ligands can be obtained with acceptable yields by condensing the corresponding amine and aldehyde or ketone with the mole ratio 1:2. The four Mn(III) Schiffbase compounds could be obtained by referencing the procedure in the previous literature [36,37].

*Caution!* KCN is hazardous and hypertoxic. Metal complexes containing perchlorate are possibly explosive sothese materials should be treated with great caution.

### 2.2. Synthetic General Procedure for the Hqxcq Ligand and the Complexes 1–5

The Hqxcq ligand [38]: To the solution of 2-quinoxalinecarboxylicacid (0.87g, 5mmol) in pyridine (2mL) was added 8-aminoquinoline (0.72 g, 5 mmol) in pyridine (2mL) at 373K. The mixture was stirred gently for 10 min, and triphenylphosphite (1.55 g, 5 mmol) was added rapidly. Then, the resultant solution was heated with stirring in a water bath for 8 h. On cooling of the mixture, a dark yellow solid resulted, with a weight of 1.2 g (80%). An analytical sample was twice recrystallized from CH_2_Cl_2_ to give yellow needles. The recrystallized product was dissolved in CH_2_Cl_2_ and purified over silica gel withthe mixture of petroleum ether and ethyl acetate [3:1, v:v] as the eluent. The major band was collected, giving the bright yellow colormicrocrystallinetarget compound (Figure S1, Supplementary Materials) with a yield of about 60% (meltingpoint: 190–192 °C), which was structurally characterized withInfrared Spectrum and Nuclear Magnetic Resonance (Figures S2 and S3).

^1^H NMR (400 MHz, CDCl_3_, δ ppm): δ−12.25 (m, 1H), 9.984 (m, 1H), 9.04–9.00 (m, 2H), 8.40–8.38 (m, 1H), 8.24–8.21 (m, 2H), 7.92–7.90 (m, 2H), 7.65–7.60 (m, 2H), 7.53 (q, J = 4 Hz, 1H).

^13^C NMR (100 MHz, CDCl_3_, δ ppm): δ 161.7, 148.9, 144.1, 144.0, 144.0, 140.5, 139.3, 136.4, 134.1, 131.8, 130.9, 130.3, 129.5, 128.2, 127.4, 122.6, 121.8, 117.1.

Complex 1: The red solution, which was formed by mixing Hqxcq (3.00 g, 10.0 mmol) and FeCl_3_·6H_2_O (2.70 g, 10.0 mmol)dissolved in 20 and 60 mL methanol, respectively, was refluxed for 1h before KCN (3.91 g, 60.0 mmol) diluted in distilled water (40 mL) was then added. The solution was refluxed for 8 h until the color of the solution changed into dark blue. After cooling and filtering, the filtrate was concentrated under reduced pressure to about 30 mL. [Ph_4_P]Br (4.20 g, 10.0 mmol) was poured slowly into the filtrate, resulting in the target blue crystalline solid, which was collectedby suction filtration, washed with distilled water (2 × 10 mL), and dried for 12 h at r.tunder vacuum. Dark-blue crystals used for single x-ray diffraction were obtained by slowly evaporating the methanol solution of complex 1 for about one week at 298 K.

(Ph_4_P)[Fe(qxcq)(CN)_3_]·0.5H_2_O (1): Yield: 4.5 g (57.6%). Anal. Calcd. for C_45_H_32_FeN_7_O_1.5_P: C, 69.15; H, 4.13; N, 12.54; Found: C, 69.08; H, 4.01; N, 12.67%. IR (KBr, cm^−1^): 2115 (s,νC≡N).

Complexes 2 and 3: The preparation methods for the two complexes involved a similar volatilization method. The addition of [Mn(L^1^)(H_2_O)_2_](ClO_4_) (73.5 mg, 0.1 mmol) or [Mn(L^2^)(H_2_O)_2_](ClO_4_) (60.6 mg, 0.1 mmol) in methanol:water (1:1) to complex 1 (78.1mg, 0.1 mmol) in methanol produced a brown mixture solution. The mixture was only stirred for 10min at 298 K and filtered to remove the insoluble material, if any. By slowly evaporating the filtrate with nodisturbance for about one week, single crystals suitable for x-ray structural analysis wereobtained by filtration, then washed with the cooled methanol, and dried under air.

[Fe(qxcq)(CN)_3_][Mn(L^1^)(H_2_O)(CH_3_OH)]ClO_4_·1.5MeOH·0.5H_2_O (2): Yield: 46.8 mg, 53.9% (basedon the manganese compound). Anal. Calcd. for C_59.5_H_58_Br_4_ClFeMn_2_N_11_O_18_: C, 41.16; H, 3.37; N, 8.87. Found: C, 41.03; H, 3.28; N, 8.97%. IR (KBr cm^−1^): 2153, 2120(s, νC≡N), 1620(vs, νC=N).

{[Fe(qxcq)(CN)_3_][Mn(L^2^)]}_2_·0.5H_2_O (3): Yield: 36.9 mg, 42.6%. Anal. Calcd. for C_82_H_71_Fe_2_Mn_2_N_18_O_12.5_: C, 60.82; H, 4.42; N, 15.57. Found: C, 60.71; H, 4.33; N, 16.41%. IR (KBr cm^−1^): 2125, 2158 (s, νC≡N), 1627 (vs, νC=N).

Complex 4 and 5: The preparation methods for the two complexes were similarto the three layers diffusion method. 1(78.1mg, 0.1 mmol)solved in 5 mL water and methanol (1:1) was laid in the bottom of the single tube, above which a mixture of H_2_O and MeOH (1:3) was added dropwise.Then, the methanol:acetonitrile (1:1) solution containing [Mn(L^3^)(H_2_O)_2_]ClO_4_ (47.7mg, 0.1 mmol) or [Mn(L^4^)(H_2_O)_2_]ClO_4_ (57.3mg, 0.1 mmol) was cautiously added to the top of the buffer layer. Single crystals with a suitable dimension used forx-ray analysis were produced about two weeks later.

[Fe(qxcq)(CN)_3_][Mn(L^3^)] (4): Yield: 41.1 mg, 57.8%. Anal. Calcd. for C_33_H_29_FeMnN_9_O_3_: C, 55.79; H, 4.11; N, 17.44. Found: C, 55.67; H, 4.02; N, 17.32%. IR (KBrcm^−1^): 2118, 2155(s, νC≡N), 1625 (vs, νC=N).

[Fe(qxcq)(CN)_3_][Mn(L^4^)]·1.5MeOH·0.5CH_3_CN·0.25H_2_O (5): Yield: 49 mg, 55.5%. Anal. Calcd. for C_43.5_H_41_FeMN_9.5_O_4.75_: C, 59.13; H, 4.68; N, 15.06. Found: C, 59.01; H, 4.78; N, 15.15%. IR (KBr cm^−1^): 2120, 2157 (s, νC≡N), 1627 (vs, νC=N).

### 2.3. Single-Crystal X-Ray Diffraction and Structural Refinement

For the reported complexes **1**–**5**, the corresponding single-crystal for x-ray analyses with a suitable sizewas mounted ona glass rod, and the crystal diffraction data were collected by using the ω scan mode on a Bruker APEX2 CCD diffractometer with a Mo Kα sealed tube (λ = 0.71073 Å) at room temperature. All structures were resolved using the direct method and further expanded employing Fourier difference techniques with the SHELXTL-2018 program package. All of the non-hydrogen atoms were anisotropically refined with anisotropic displacement coefficients. Hydrogen atoms were distributed to the isotropic displacement coefficients with U(H) =1.2 or 1.5U(C), and the coordinates were permitted to ride on the corresponding carbons utilizing SHELXL-2018, except for some solvent H atoms. The H atoms from the solvent molecules were isotropically refined with fixed U values, during which the DFIX command was utilized for the purpose of rationalizing the bond parameter. The supplementary crystallographic data for the reported complexes **1**–**5** were deposited in The Cambridge Crystallographic Data Center (CCDC) with the number 1902326-1902330, which is freely available from the Cambridge Crystallographic Data Centre [39]. The crystal parameters, data collection, and refinement are summarized in detail in Table S1 (Supplementary Materials). Some important bond parameters for complexes **1**–**3** and **4**–**5** are listed in Tables S2 and S3, respectively. The measured and calculated powder x-ray diffraction (PXRD) patterns for compounds **1**–**5** are given in Figures S4–S8 (Supplementary Materials). The calculated and experimental diffraction peaks were basically in the same position, demonstrating the phase purity of these compounds.

### 2.4. Physical Measurements

Elemental analysis for C, H, and N were carried out by using an Elementary Vario El and the infrared spectra were measured by employing KBr disks on a Shimadzu FTIR-8600 spectrophotometer. Magnetic susceptibilities were measured by a Quantum Design SQUID MPMS-XL magnetometer, where the *dc* measurements were collected within the temperature range 2–300 K and the field range of 0–50 KOe. All of the experimental susceptibilities were corrected for the diamagnetism of the constituent atoms (Pascal’s tables).

## 3. Results and Discussion

### 3.1. Preparation and General Characterization

The Mn^III^ quadridentate Schiffbase compounds with always sizable anisotropic characteristics have been widely utilized to synthesize the CN-bridged magnetic compounds not only due to their easy preparation but also to the big spin (S = 2) and the intrinsic Jahn–Teller effectalong the axial direction [36]. Reactions of the four *quasi* planar tetradentate Schiffbase ligands based manganese(III) compounds with the new *mer*-tricyanoiron(III) building block provided four CN-bridged heterobimetallic compounds with different structural conformations, for which the corresponding synthesis scheme for the organic ligand and complexes **1**–**5** are provided in Scheme S1, indicating that the structure of the different Schiffbase has an obvious influence on the structural type of the target cyano-bridged complexes. Additionally, the obtained results also imply that the *mer*-tricyanoiron(III) precursor [Fe(qxcq)(CN)_3_]^−^ is more favorable for constructing a low-dimensional molecular magnetism system.

The IR spectroscopy characterization (Figures S9–S13) for the CN-bridged Fe(III)-Mn(III) complexes**2**–**5** as well as PPh_4_[Fe(qxcq)(CN)_3_]·0.5H_2_O werecarried out. In the infrared spectra of the cyano precursor, the absorption positioned at about2115 cm^−1^ was distributed to the uncoordinated cyanide group. For complexes **2**–**5**, two sharp peaks originating from the CN group stretching vibration could be observed within 2120–2160 cm^−1^, implying the existence of both bridging and non-bridging cyano groups in these complexes.

### 3.2. Crystal Structure for Complexes **1**–**5**

**Figure 1:**
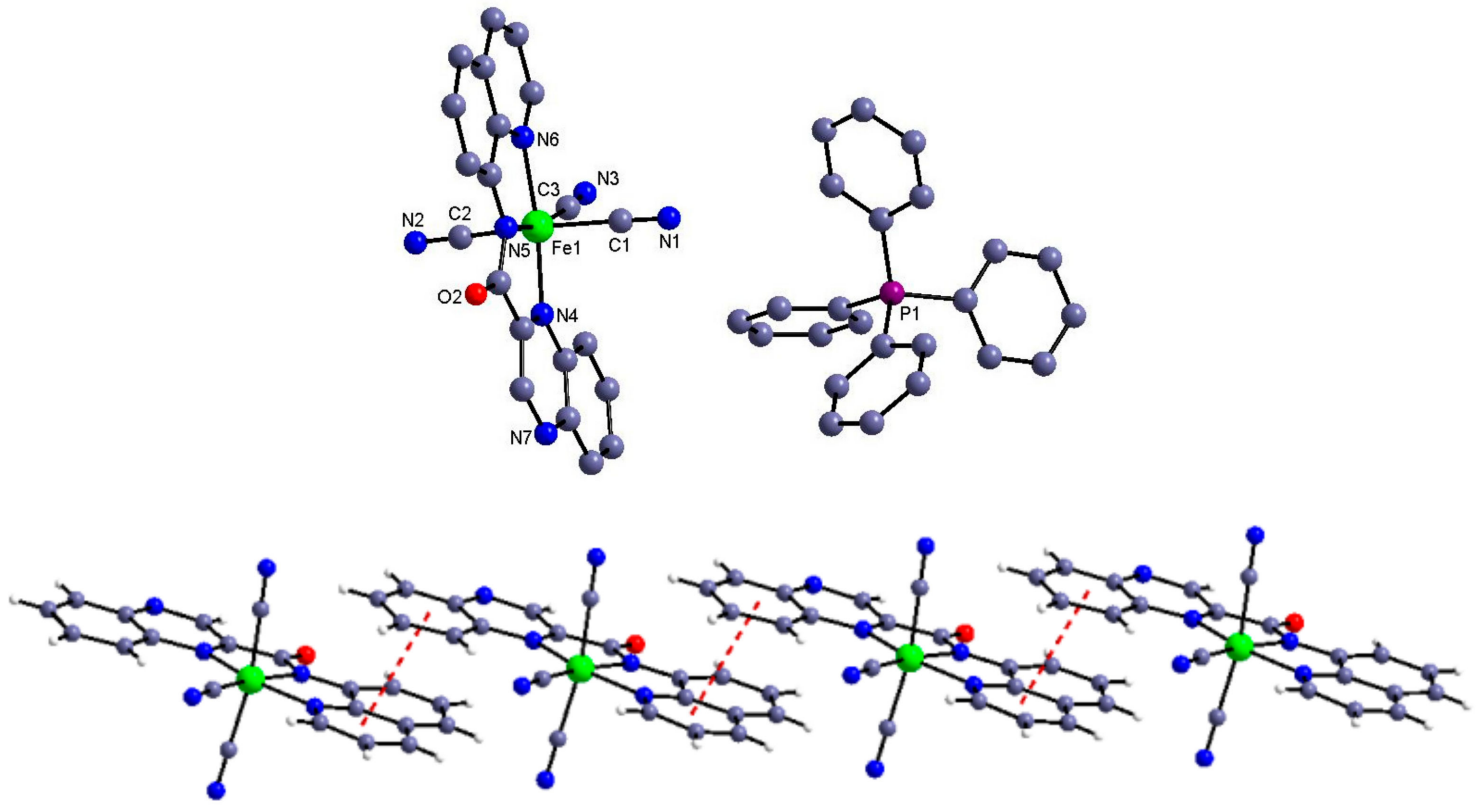
Molecular view of **1** with the selected atom-labeling scheme (**top**) and the one-dimensional supramolecular structure resulted from the intermolecular π–π interaction (**bottom**). The solvent water molecule has been omitted for clarity.

Complex 1. The crystal structure of complex **1** is comprised of PPh_4_^+^ cations and [Fe(qxcq)(CN)_3_]^−^ anions (Figure 1). The Fe atom is complexed by three CN groups and three N donors of qxcq in a meridional configuration, forming octahedral coordination geometry. The Fe–C bonds are in the normal range compared with other reported *mer*-tricyanoiron(III) precursors [24,25,26,27,28,29,30,31,32,33,34]. One of the three Fe–N_amide_ bond lengths with the value 1.945(5) Åwas slightly shorter than the other ones (2.004(5)–2.060(5) Å) relevant with qxcq, which can be attributed tothe strong σ-donor influence of the deprotonated amide ligand. Under the help of the intermolecular π–πinteraction from the aromatic rings of the amide ligands, the tricyanoiron(III) anion can be assembled into a supramolecular one-dimensional structure (Figure 1).

**Figure 2:**
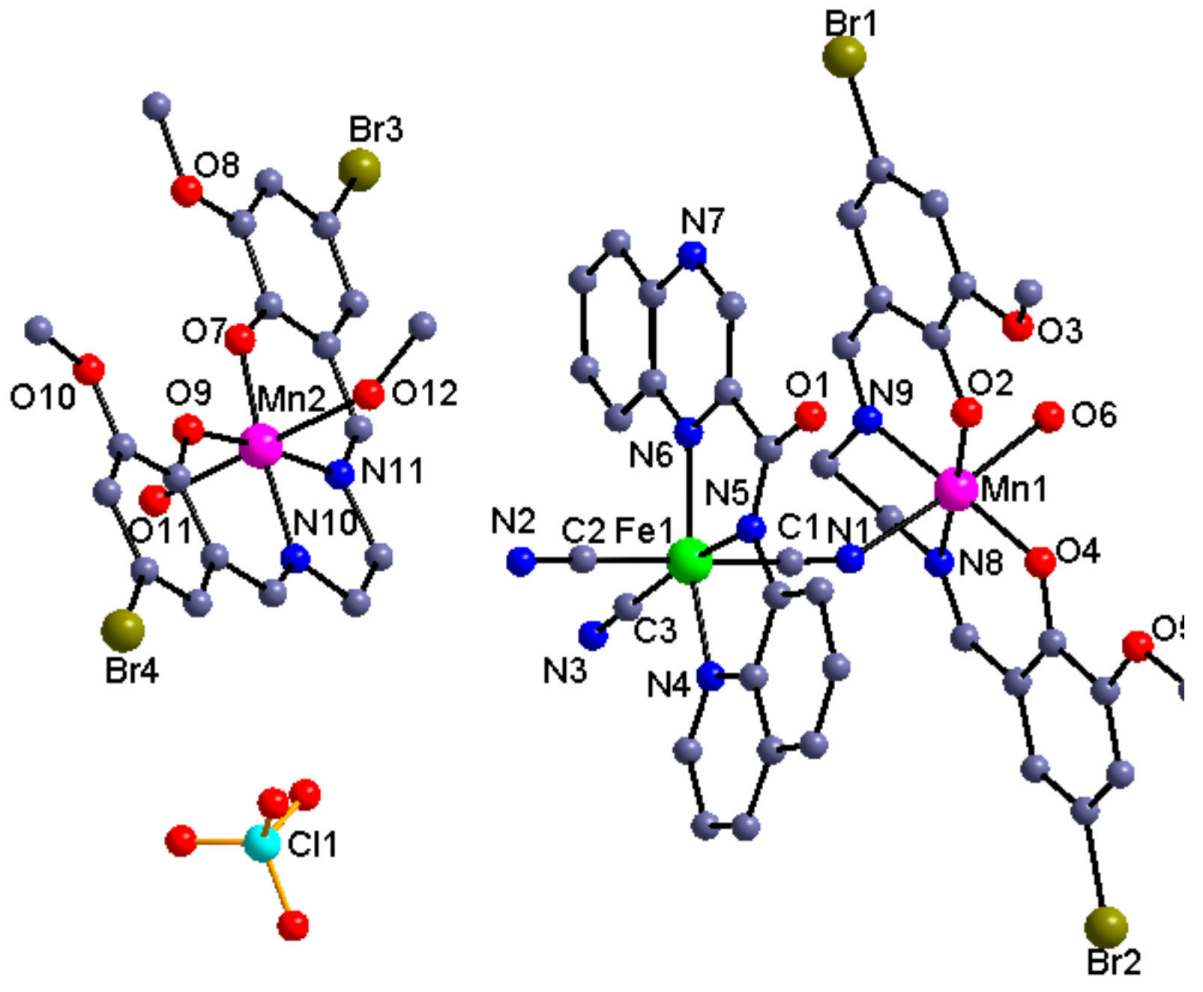

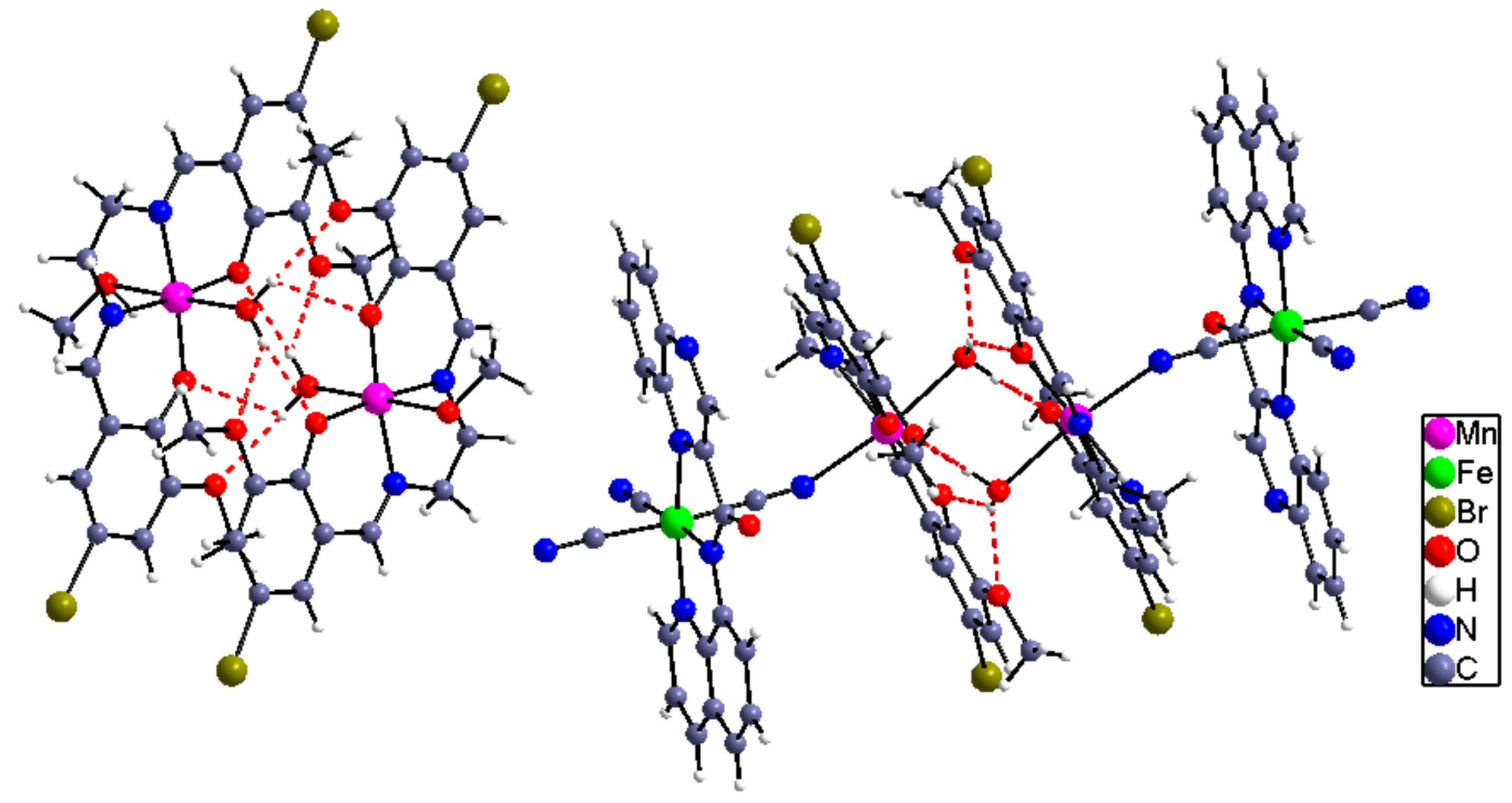
Molecular view of **2** with the selected atom label scheme and its H-bond interaction resulting supramolecular dimeric structure. The solvent contents have been omitted for clarity.

**Figure 3:**
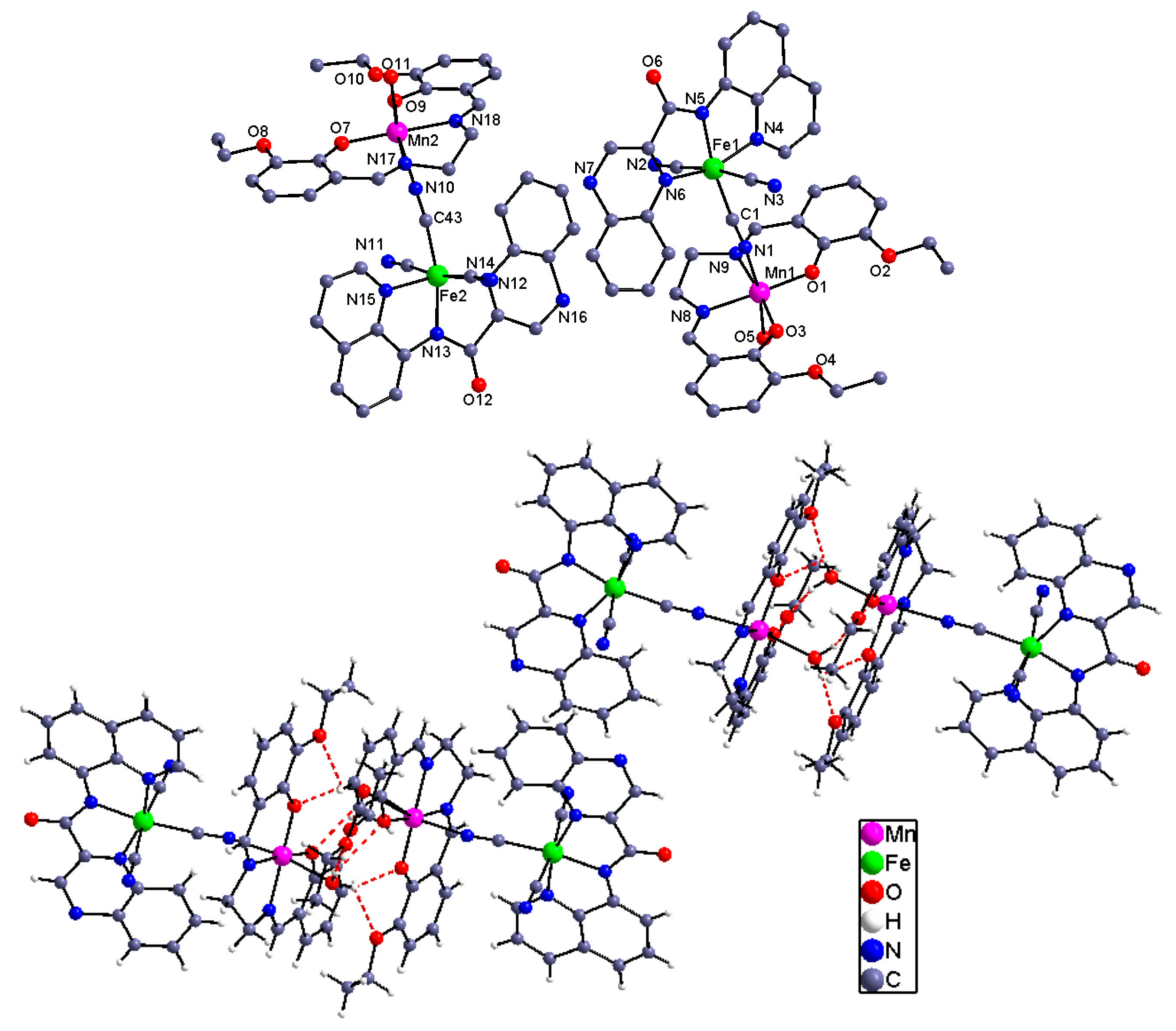
Molecular view of **3** with the selected atom-labeling scheme and its H-bond interaction resulting supramolecular dimeric structure. The solvent contents have been omitted for clarity.

Complexes 2 and 3. Figure 2 and Figure 3 depict the molecular structure and the corresponding dimeric structure constructed by the intermolecular H-bond coactions for complexes 2 and 3, respectively. Complexes2and3are crystallized in a triclinic *P-1*space group. Complex 2 contains a cyanide-bridged binuclear unit and an additional co-crystallized mononuclear manganese(III) Schiffbase unit [Mn(L^1^)(H_2_O)(CH_3_OH)]ClO_4_, while complex 3 belongs to the pure cyanide-bridged binuclear structure containing two independent [Fe(qxcq)(CN)_3_][Mn(L^2^)(H_2_O)] units. Unlike complex 2, where the dimeric unit is constructed from the cationic [Mn^III^(L^1^)]^+^ part and the anionic precursor [Fe^III^(qxcq)(CN)_3_]^−^ islinked by one of two *trans-*cyanide groups, the [Mn^III^(L^2^)(H_2_O)]^+^ cation in complex 3 is coordinated by the equatorial cyanide group of the *mer-*tricyanoiron(III) precursor. The difference in the structural composition and the coordination mode could be assigned to the different steric effect (MeO– or EtO–) of the Schiff base ligand. For both of these complexes, the structural extension is blocked by the water molecule coordinated to one of the axial positions of the manganese(III) Schiffbase. Similar to that in compound 1, the coordination geometry for the Fe(III) in the crystal structures of 2 and 3 can also be defined as a distorted octahedral involved in a C_3_N_3_ unit. A similar reason to that in complex 1 can be used to explain the shorter Fe–N_amide_ bond distances of [Fe1–N5 = 1.906(5) Å] and [Fe1–N5 = 1.945(8) Å] for 2 and 3 in comparison to the other Fe–N lengths ranging from about 1.98–2.03 Å [25,27,29,30].

The Mn(III) ion in the cyanide-bridged entity is six-coordinated with the four equatorial positions bonded by a N_2_O_2_ unit from the Schiff base and the two axile ones from the N atom of the CN_bridging_ group and the O atom of the solvent molecule. For all of the Mn(III) ions in complexes 2 and 3, the Jahn–Teller distortion was clear with an elongated octahedralproved by the shorter equational Mn–N(O) distances [mean Mn–N(O) = 1.989, 1.882 Å] than those of the axial Mn–N_cyanide_/O_solvent_bond lengths [2.253(5), 2.308(3) Å and 2.248(6), 2.296(5)Å]. The Mn–N≡C angles corresponding to 153.4(4)° for 2 and 160.2(7)°for 3 showed a moderate difference and deviated obviously from the linear conformation. The intramolecular Fe–Mn distances through the cyano linkage were 5.169 Å in 2and 5.259, 5.281 Å in 3, respectively. Both of the cyanide-bridged binuclear entity and the monocnuclear unit can be self-assembledby the coordinated solvent molecule from one complex and the free O_4_unit from the adjacent complex, therefore giving the supramolecular dimerized structure. Furthermore, the dimeric moieties are linked into a one-dimensional supramolecular architecture by the π–π stacking interactions between the qxcq ligands.

**Figure 4:**
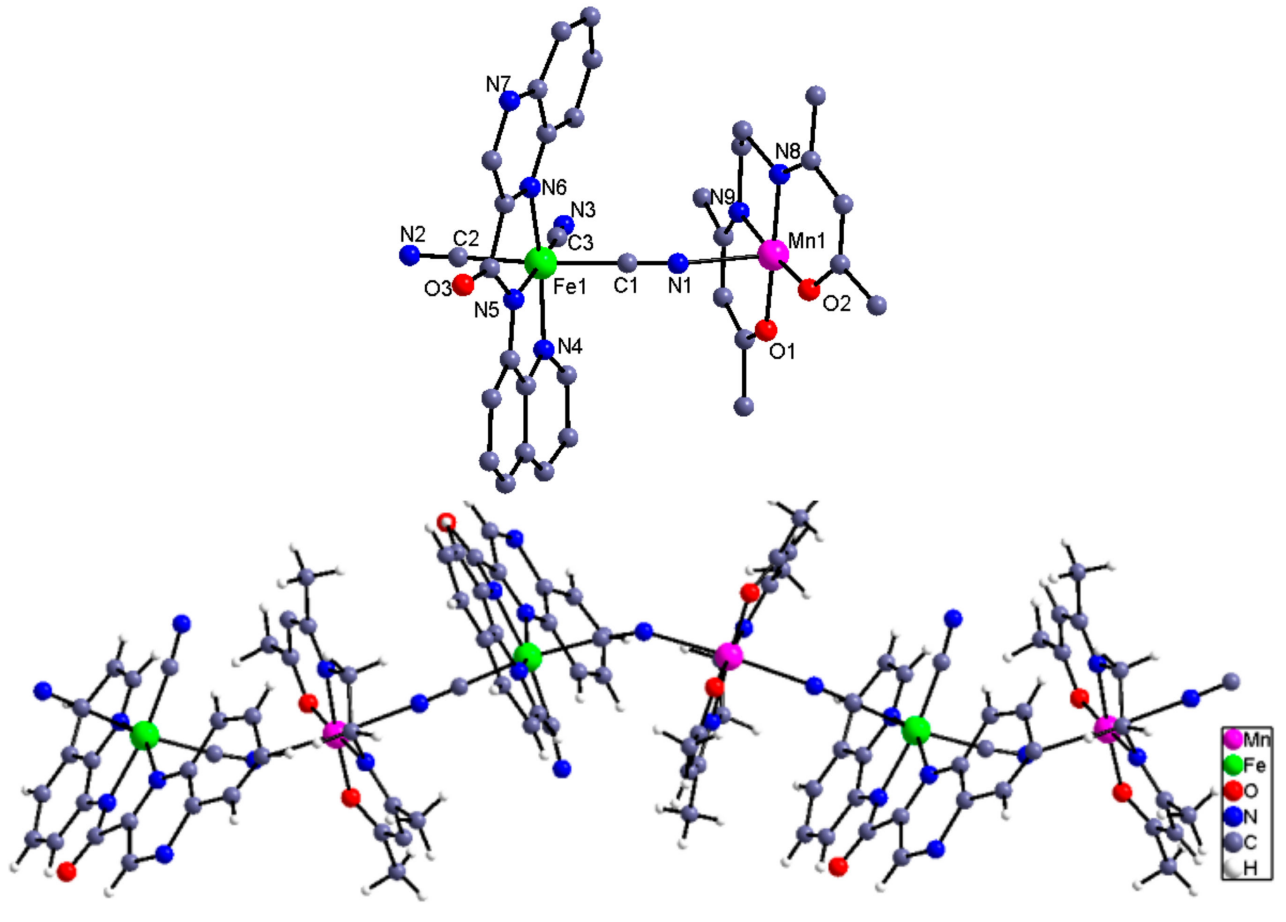
The independent binuclear and 1D structure of complex **4**.

**Figure 5:**
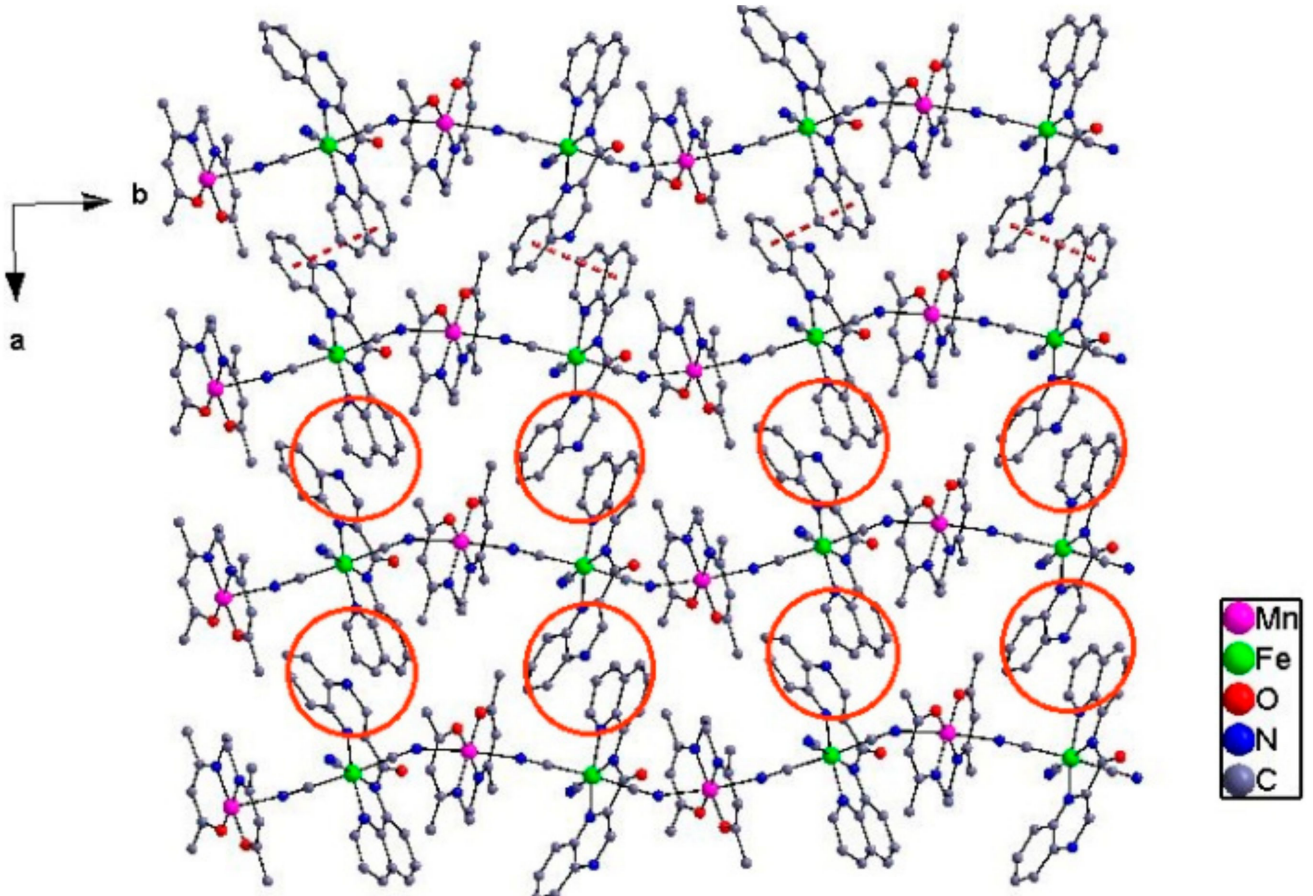
The cell packing diagram showing the π–π interaction along the*a* axis for complex **4**.

**Figure 6:**
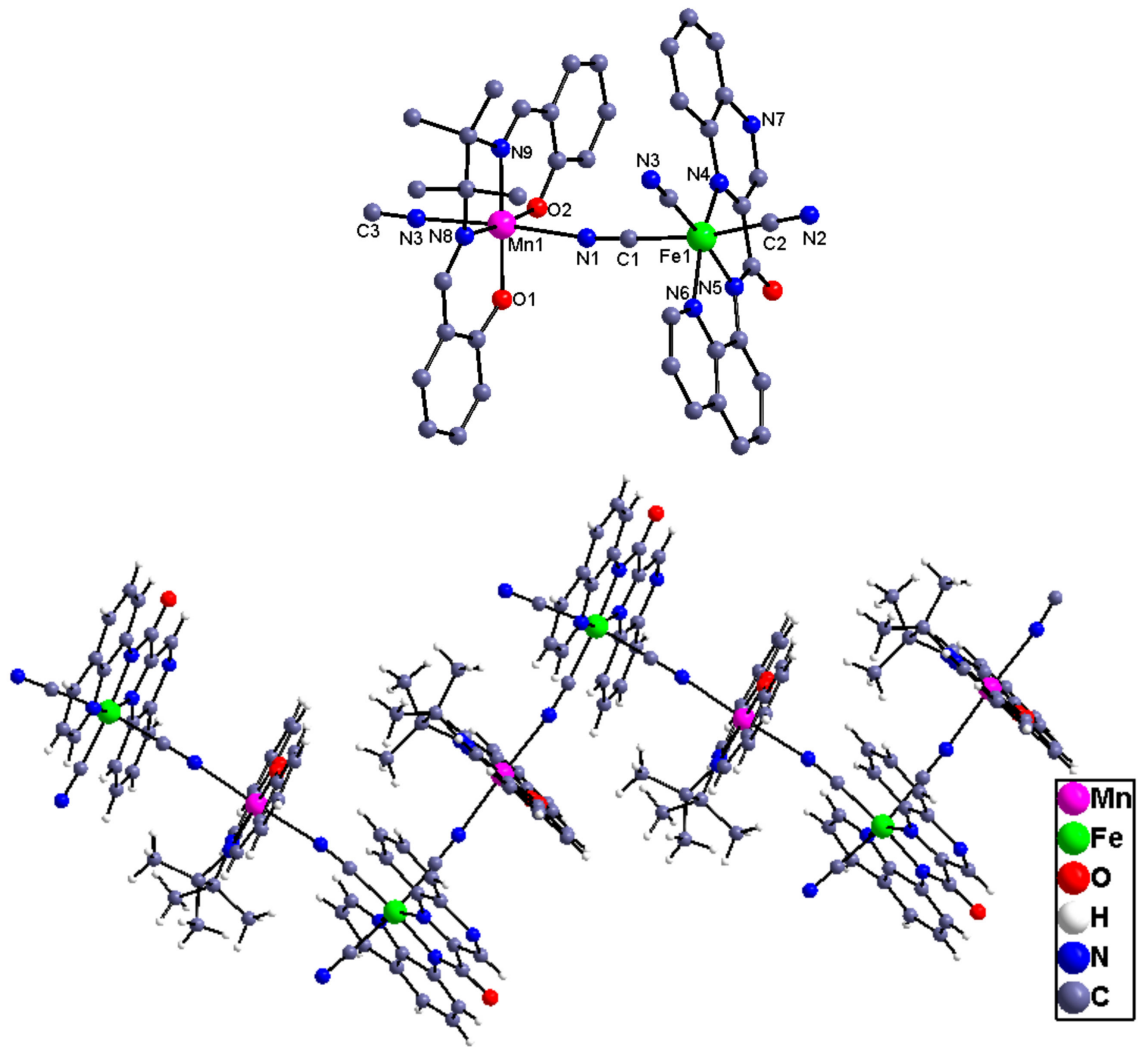
The independent binuclear and 1Dchain structure of complex **5**. The solvent content has been omitted for clarity.

Complexes **4** and **5**. Figure 4 and Figure 5 show the independent binuclear, one-dimensional structure and the cell packing diagram presenting the π–π interaction along *a* axis for complex **4**. For complex 5, its binuclear and one-dimensional structure is given in Figure 6. Complexes **4** and **5**, which crystallize in the monoclinic *P2(1)/n* space group and contain four independent units, can be structurally categorized as a 1D chain comprised of the alternated [Fe^III^(qcq)(CN)_3_]^−^ anion and the Schiff base Mn(III) cation. Within the chain, each of the Schiff base Mn^III^ cations connects two [Fe^III^(qcq)(CN)_3_]^−^ anions in *trans* arrangement, while each [Fe^III^(qcq)(CN)_3_]^−^ anions coordinate axially to two Mn^III^ (Schiff base) cations in*trans*-mode for 4 and *cis*-mode for 5, therefore resulting in the linear conformation and zig-zag arrangement for complexes 4 and 5, respectively. The structural parameters around the Fe atom were also quite similar to those in complex 1, indicating that the coordinating to the manganese Schiff base had no marked influence on the coordinationgeometry of the Fe(III) ion.

For the manganese Schiff base part, the Mn^III^ ion waslocated in the highly elongated octahedron (Jahn–Teller effect), where thefour equatorial sites were occupied by four N/O atoms [Mn–N/O distances: 1.874(4)to 1.985(4) Å] from the Schiff base and the two axial positions were complexed by two N atoms [Mn-N_cyanide_ distances: 2.284(9) to 2.326(4) Å] from the CN groups of two neighboring [Fe^III^(qcq)(CN)_3_]^−^units. With respect to the Mn–N≡C bond angles in the bridging pathways for these two complexes, an obvious difference could be found. These types of bond angles deviatedobviously from the linear conformation with values of159.0(9) and 154.8(9)° in complex 4, while the corresponding angles remained most linear with the values 172.4(4) and 173.4(4)° in complex 5, demonstrating that the structure of the Schiff base manganese(III) and the coordination mode play important rolesin the Mn–N≡C bending. The linear chain in complex 4runs with intra-chain Mn…Fe distances of 5.205 Å corresponding to 5.408 Å in the zig-zag chain running along the *b*axis. The shortest inter-chain metal…metal separationswere 8.486 Å for complex 5, which was obviously shorter than the corresponding distance in complex 4 with the valueof 8.886 Å. It should be pointed out that either of the linear or the zigzag chains could be stacked in parallel by the π–π stacking interaction between the [Fe^III^(qcq)(CN)_3_]^−^moieties, leading to the 2D supramolecular sheet, where the solvent contents (for complex 5) were filled in the channels along the *a* or *c* axial.

### 3.3. Magnetic Properties of Complexes **2**–**5**

**Figure 7:**
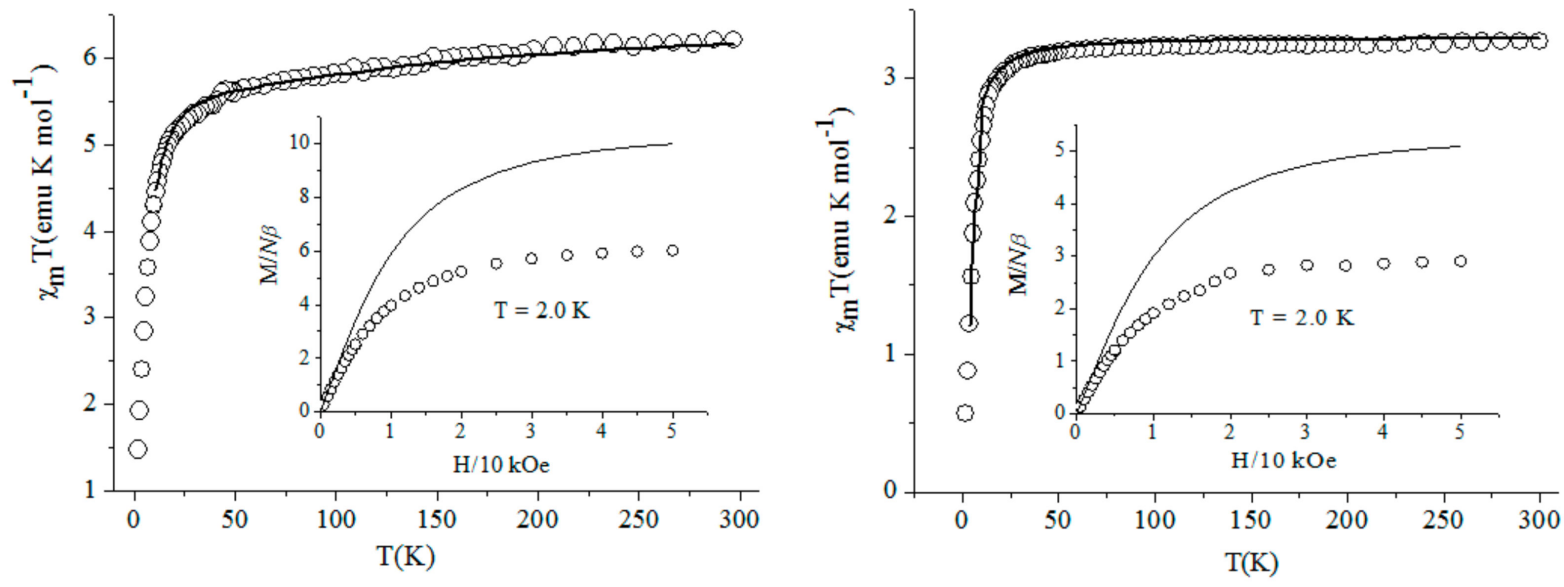
The magnetic property of complexes **2** and **4**.

**Figure 8:**
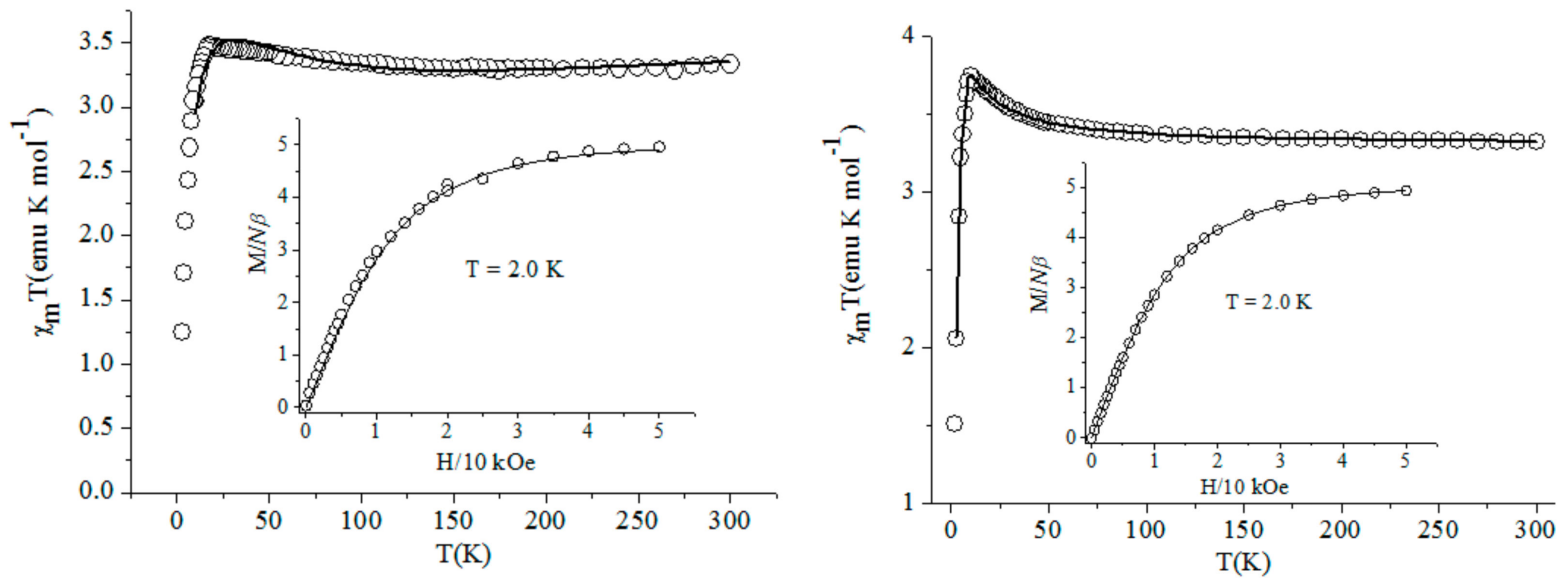
The magnetic property of complexes **3** and **5**.

The temperature dependent magnetic susceptibility with the form of per Fe^III^Mn^III^_2_ for complex 2 and per Fe^III^Mn^III^ for complexes 3–5 measured from 2–300 K under the applied magnetic field of 2000 Oe using the corresponding single crystal with the quantity about 10-–0 mg are shown in Figure 7 and Figure 8, respectively. The variation trends of the χ_m_*T–T* curves for compounds 2 and 4 were similar to each other, which was also similar for complexes 3 and 5. The χ_m_*T* values at r.t. for these four complexes were about 6.16, 3.27, 3.32, and 3.31 emu·K·mol^–1^, respectively, which were basically in agreement with the spin-only value of 6.375 for the two isolated Mn(III) ions (*S* = 2), one low spin Fe(III) ion (S = 1/2) for 2 and 3.375 emu·K·mol^–1^ for one Mn(III) ion, and one Fe(III) ion for 3–5, respectively. For compounds 2 and 4, the χ_m_*T* values decreased slowly, accompanying the temperature down to about 50 K, then the χ_m_*T* values began to decrease quickly and reached their lowest value at about 1.47 and 0.56 emu·K·mol^–1^ at 2 K, respectively, primarily indicating the antiferromagnetic interaction between the Mn(III)–Fe(III) unit through the bridging cyanide group. The χ_m_*T–T* curves for complexes 3 and 5 presenteda basically contrary change tendency to the above two compounds. By decreasing the temperature, the χ_m_*T* value for these two complexes remained almost constant until 75 K, then began to increase smoothly and attained their maximum value at about 3.50 emu·K·mol^–1^ for 3 and 3.72 emu·K·mol^–1^ for 5, respectively, and then decreasedwith high speed to the minimum value of about 1.50 and 1.24 emu·K·mol^–1^for 3 and 5, respectively, at 2 K. The magnetic behavior of the complexes at very low temperature can probably be due to the intermolecular anti-ferromagnetic interaction resulting from the inter-molecular weak interactions and/or the zerofieldsplitting (ZFS) effect of the Mn(III) ion. The described change tendency for the χ_m_*T–T* curves above primarily implied the antiferromagnetic and ferromagnetic coupling between the cyanide-bridged Mn(III) ion and Fe(III) ion in complexes 2 and 4 and 3 and 5, respectively.

The magnetic susceptibilities for complexes 2–5 obey the Curie–Weiss law well in 10–300 K. The obtained negative Weiss constant *θ*= −5.2 K, Curie constant *C*= 6.26 emu·K·mol^–1^ for 2, *θ*= −2.65 K, *C*= 3.30 emu·K·mol^–1^ for 4, positive *θ*= 0.65 K, *C*= 3.33 emu·K·mol^–1^ for 3, and *θ*= 1.35 K, *C*= 3.32 emu·K·mol^–1^ for 5, further confirming the overall antiferromagnetic or ferromagnetic interaction between the Mn(III) ion and Fe(III) ion in these complexes. The insets of Figure 7 and Figure 8 show that the field-dependent magnetization was measured up to 50 kOe at 2 K for the corresponding complexes. The magnetization for all complexes increasedquickly up to about 10 kOe. After that, the curve becamecomparatively even and gradually reached their largest value. The values at 50 kOe were 6.1 and 2.9 *Nβ* for complexes 2 and 4, which were significantly lower than the expected 9 and 5 *Nβ*for the uncoupled two or one high spin Mn(III) ion and one low spin Fe(III) ion based on *g* = 2.0, proving again the existence of overall antiferromagnetic coupling in complexes 2 and 4. The maximum field-dependent magnetization value for complexes 3 and 5 were about 4.9 *Nβ*, very close to the saturated value 5 *Nβ*corresponding to the spin state *S* = 5/2 from the high-spin Mn(III) ion andlow-spin Fe(III) ion, further revealing the intramolecular ferromagnetic interaction in them.

The theoretical magnetism analysis for complexes 2–5 was carried out according to their structural types.Complex 2 can be structurally considered as the cyanide-bridged binuclear entity by adding an isolated free Mn(III) ion compound, while complex 3 belongs to only a pure cyanide-bridged binuclear structure. Basedon the dinuclear Mn^III^-Fe^III^ (*S* = 2 and 1/2) model, the magnetic susceptibilities for these two compounds can be simulated by the following expression originatingfrom the isotropic exchange spin Hamiltonian *Ĥ* = −2*J*Ŝ_Fe_Ŝ_Mn_. However, an additional isolated Mn(III) ion (*χ_Mn_ = Ng^2^β^2^Ŝ_Mn_(Ŝ_Mn_ +1)/3kT, S = 2*) needs to be included for complex **2**.

*χ_d_ = Ng^2^β^2^(35exp(2J/kT) + 10exp(-3J/kT))/2kT(6exp(2J/kT) + 4exp(-3J/kT))* (1)

χ_m_ = χ_d_/(1-χ_d_(2zJ’/ *Ng^2^β^2^)* (2)

The magnetic data for these two compoundswere fitted in the temperature range of 10–300 K by introducing the mean-field approximation (*zJ’*), accounting for the effects other than the intramolecular magnetic coupling. The obtained best-fit parameters were *J* = −5.56 cm^−1^, *g* = 1.99, *zJ’* = −0.81 cm^−1^and *J* = 2.15 cm^−1^, *g* = 1.98, *zJ’* = −0.58 cm^−1^for complexes **2** and **3**, respectively. The negative *zJ’* values indicate that antiferromagnetic interactions occur between dimeric units, which are related to the presence of the intermolecular H-bonds. These results are basically comparable to those values found in the cyanide-bridged Fe^III^Mn^III^ polynuclear complex previously reported [35].

**Scheme 2:**
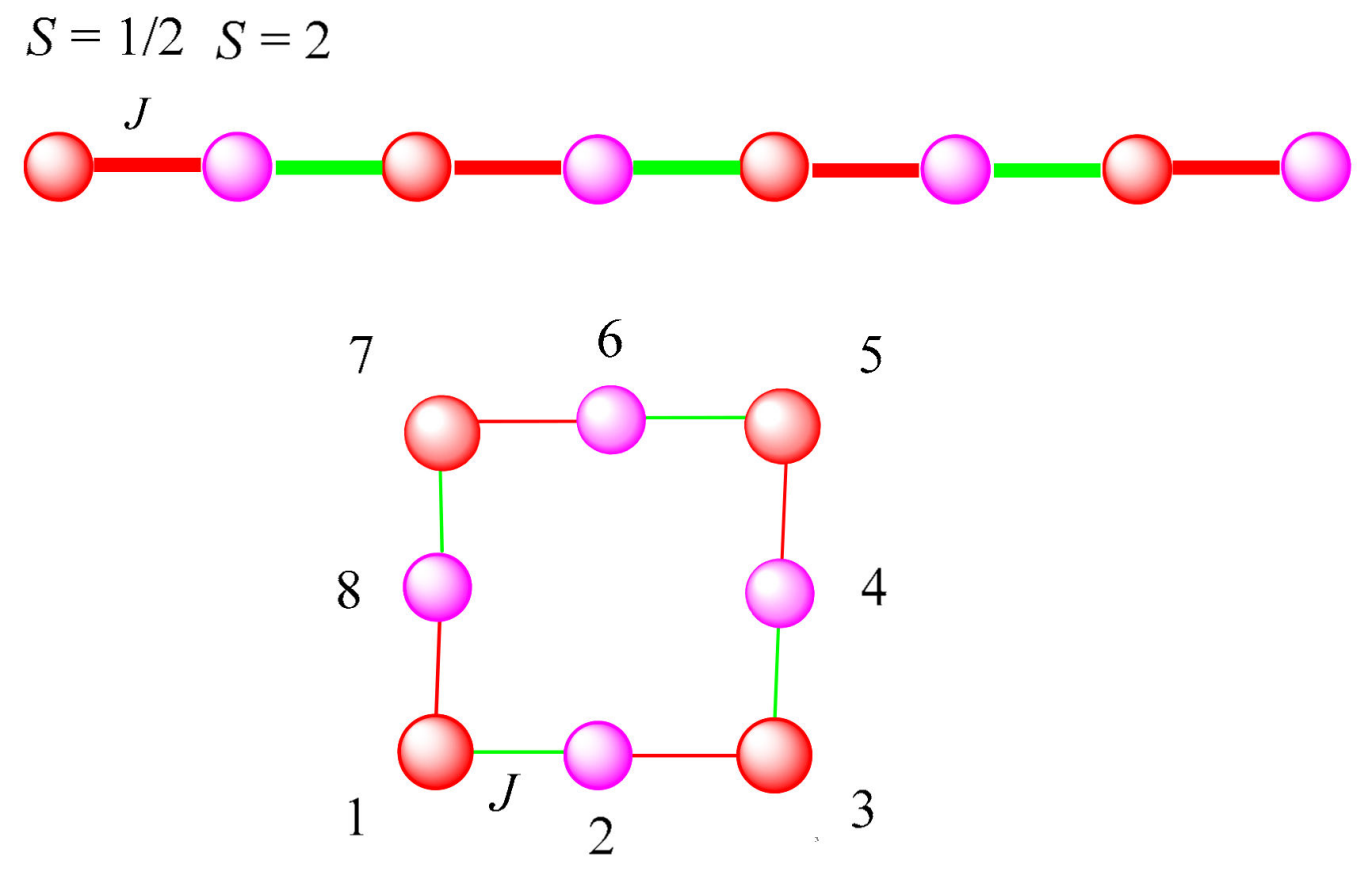
The model used to simulate the magnetic property of complexes **4** and **5**.

The magnetic data for the two one-dimensional chain-like complexes 4 and 5 werefitted by the Hamiltonian *Ĥ* = *Ĥ_ex_* + *Ĥ_anis_* + *gβĤŜ*, where the first term corresponds to the isotropic interactions (the magnetic couplings through the single cyano bridge). The second term deals with the local anisotropy of the high spin Mn(III) ion, and the last one is the Zeeman effect. Considering that the two independent Fe(III)–CN–Mn(III) linkages in these two complexes were not markedly different to the two Mn–N≡C bond angles 154.7(10), 158.5(10)° in 4 and 173.4(4), 172.4(4)° in 5, respectively, these two compounds could be treated as a chain containing alternative spins 1/2 and 2 with approximately one exchange coupling J (Scheme 2). In this case, the magnetic susceptibilities of the two 1D chains could be fitted and calculated rationally on the basis of a closed loop model comprised by four pairs of 1/2–2 spin pairs. The simulation was done by numerical matrix diagonalization techniques employing a Fortran program [40]. The best fit parameters *J* = −2.56 cm^−1^, *D* = −1.72 cm^−1^, *g* = 2.03 and *R* = ∑(χ_obsd_*T*-χ_cald_*T*)^2^/∑(χ_obsd_*T*)^2^= 6.45 × 10^−5^ for 4 and *J* = 4.01 cm^−1^, *D* = −1.82 cm^−1^, *g* = 2.01 and *R* = 1.34 × 10^−5^ for 5, respectively.

### 3.4. Magneto–Structural Correlation

As has been proven, due to the strict orthogonality of the magnetic orbitals of [*d*_xy_/*d*_xz_/*d*_yz_]^1^ in iron(III) vs. [*d*_z_^2^]^1^ in manganese(III) and the overlap between the corresponding *d*_xz_, *d*_yz_, *d*_xy_ orbitals, ferromagnetic or antiferromagnetic interaction could both be possible inCN-bridged Fe^III^–Mn^III^ systems. Two factors contributed mainly to the magnetic coupling in the CN-bridged Fe^III^–Mn^III^ complex, where one was the magnetic orbital of the low-spin Fe^III^ ion with a t_2g_^5^e_g_^0^ electronic arrangement and the other was the bending of the Mn–N≡C_bridge_. The electronic arrangement of the low-spin Fe(III) ion is t_2g_^5^e_g_^0^, where the magnetic orbital employed to describe the single electron ([*d*_xy_/*d*_xz_/*d*_yz_]^1^) is sensitive to the coordination environment induced electronic effects such as the location and the number of the CN groups bonded to the Fe^III^ center. For the *mer*-tricyanoiron(III) types precursors, the DFT calculation has theoretically revealed that the main magnetic orbital may be *d_xz_* residing on the tricyano plane [35], resulting in the possibility ofdelocalizing the electron concentration on the π-pathway of the CN group, particularly on the N_CN_ atom bonded to the manganese(III) ion. Therefore, due to the orthogonality or overlap of the magnetic orbitals, *d_xz_*(Fe^III^) vs. {(*d_xz_*, *d_yz_*, *d_xy_*)*^3^*(*d_z_^2^*)^*1*^}(Mn^III^),it is both possible that the interaction through the single cyanide linkage can lead to ferro- and antiferro-magnetic coupling. Previous reports have shown that the almost always antiferromagnetic coupling in *mer*-tricyano-based Fe^III^-Mn^III^ complexes containing the always relatively smaller Mn–N≡C_bridge_ bond angles, which is in the favor of resulting in the overlap of the magnetic orbital. It is also apparent that the bigger the Mn–N≡C_bridge_ bending, the lower the magnetic orbital overlap and therefore increase the possibility of magnetic orbital orthogonality. As a consequence, the switch of the magnetic interaction nature between Fe^III^–Mn^III^ through the cyanide bridge from antiferromagnetic to ferromagnetic can open once the Mn–N≡C angle increases to some extent, which can be further confirmed by the present results in complexes **3**, **4** and also in one of the recently reported Fe^III^–Mn^III^ compounds basedon [Fe(qcq)(CN)_3_]^−^ with the Mn–N≡C angle over 170° [32].

## 4. Conclusions

In summary, a new *mer*-tricyanometallate wasfirst prepared and structurally characterized, and based on which and the different Schiff base manganese(III) compounds, a new series of cyanide-bridged magnetic complexes were obtained. Single crystalx-ray diffraction analysis revealed that their different structural types belonged to discrete polynuclear clusters and 1D infinite linear or zig-zag coordination polymers, which could be further dimerized by the intermolecular hydrogen bond interactions and assembled into a 2D supramolecular structure by the inter-chain π–π interactions, respectively, indicating that the structure of the Schiff base ligand could play an important rolein the structure of the target cyanide-bridged complexes. Systematic magnetic study on all complexes with the combination of the magneto–structural correlation disclosed the antiferromagnetic coupling in complexes **2** and **5** with smaller Mn–N≡C_bridge_ bond angles between the cyanide-bridged Fe^III^–Mn^III^, while the ferromagnetic magnetic interaction in the other two complexes containing the larger Mn–N≡C_bridge_ bond angle, which for the latter has been seldom been previously reported with regard to the *mer*-tricyano-based Fe(III)-Mn(III) magnetic assemblies. The results reported here offer meritorious information for the rational design of new*mer*-tricyano-containing precursors used to constructmolecular magnetism systems in coordination chemistry with various structural types and different magnetic coupling nature. The obtained new series of bimetallic complexes can further enrich the members of the cyanide-bridgedfamily, which are very helpful for thoroughly discovering the magneto–structural correlation in this field from the molecular magnetism perspective.

## Supplementary Materials

Supplementary Materials: The following are available online at https://www.mdpi.com/2073-4360/11/10/1585/s1, Figure S1: The image for the microcrystalline yellow color Hqcxq ligand under the microscope. Figure S2: The IR of the Hqcxq ligand. Figure S3–S7: The calculated and measured XRD for the complexes **1**–**5**. Figure S8–S12: The IR of the complexes **1**–**5**. Scheme S1: The synthesis scheme for the organic ligand and the complexes **1**–**5**. Table S1: Details of the Crystal Parameters, Data Collection, and Refinement for Complexes **1**–**5**. Table S2: Selected bond lengths (Å) and angles (deg) for **1**–**3**. Table S3: Selected bond lengths (Å) and angles (deg) for **4**–**5**.
