# Supplementary material for: Various Structural Types of Cyanide-Bridged FeIII–MnIII Bimetallic Coordination Polymers (CPs) and Polynuclear Clusters Based-on A New mer-Tricyanoiron(III)Building Block: Synthesis, Crystal Structures, and Magnetic Properties
Source: Polymers (Basel). 2019 Sep 27;11(10):1585. doi: 10.3390/polym11101585 (PMC6835830; doi:10.3390/polym11101585)

checkCIF/PLATON report

Structure factors have been supplied for datablock(s) 1

THIS REPORT IS FOR GUIDANCE ONLY. IF USED AS PART OF A REVIEW PROCEDURE FOR PUBLICATION, IT SHOULD NOT REPLACE THE EXPERTISE OF AN EXPERIENCED CRYSTALLOGRAPHIC REFEREE.

No syntax errors found. CIF dictionary Interpreting this report

Datablock: 1

```
Bond precision:  C-C = 0.0103 A          Wavelength=0.71073

Cell:          a=9.3316(16)       b=14.805(3)      c=15.010(3)
               alpha=109.812(3)   beta=97.307(3)   gamma=99.965(3)
Temperature:   293 K
```

	Calculated	Reported
Volume	1882.5(6)	1882.5(6)
Space group	P -1	P -1
Hall group	-P 1	-P 1
Moiety formula	2(C21 H11 Fe N7 O), 2(C24 H20 P), H2 O	(C21 H11 Fe N7 O), (C24 H20 P), 0.5H2 O
Sum formula	C90 H64 Fe2 N14 O3 P2	C45 H32 Fe N7 O1.50 P
Mr	1563.19	781.59
Dx,g cm-3	1.379	1.379
Z	1	2
Mu (mm-1)	0.491	0.491
F000	808.0	808.0
F000’	809.14	
h,k,lmax	11,17,17	11,17,17
Nref	6638	6567
Tmin,Tmax	0.873,0.898	0.871,0.901
Tmin’	0.867	

```
Correction method= # Reported T Limits: Tmin=0.871 Tmax=0.901
AbsCorr = MULTI-SCAN

Data completeness= 0.989          Theta(max)= 25.009

R(reflections)= 0.0777( 4263)     wR2(reflections)= 0.2300( 6567)

S = 1.029                  Npar= 505
```

The following ALERTS were generated. Each ALERT has the format
test-name_ALERT_alert-type_alert-level.
Click on the hyperlinks for more details of the test.

Alert level B

```
PLAT420_ALERT_2_B D-H Without Acceptor       O1       --H101     .     Please Check
```

Author Response: The electronic density arround the solvent O atom are somewhat weak, making it difficultly to accurately confirm the position and the direction of the H atoms bonded.

```
PLAT420_ALERT_2_B D-H Without Acceptor       O1       --H201     .     Please Check
```

Author Response: The electronic density arround the solvent O atom are somewhat weak, making it difficultly to accurately confirm the position and the direction of the H atoms bonded.

Alert level C

```
PLAT230_ALERT_2_C Hirshfeld Test Diff for    N4       --C21      .        6.0 s.u.
PLAT230_ALERT_2_C Hirshfeld Test Diff for    N5       --C9       .        5.4 s.u.
PLAT230_ALERT_2_C Hirshfeld Test Diff for    N5       --C13      .        6.0 s.u.
PLAT230_ALERT_2_C Hirshfeld Test Diff for    C7       --C8       .        6.4 s.u.
PLAT230_ALERT_2_C Hirshfeld Test Diff for    C20      --C21      .        6.5 s.u.
PLAT232_ALERT_2_C Hirshfeld Test Diff (M-X)  Fe1      --N5       .        6.3 s.u.
PLAT234_ALERT_4_C Large Hirshfeld Difference N7       --C15      .       0.18 Ang.
PLAT234_ALERT_4_C Large Hirshfeld Difference N7       --C16      .       0.16 Ang.
PLAT234_ALERT_4_C Large Hirshfeld Difference C6       --C7       .       0.17 Ang.
PLAT234_ALERT_4_C Large Hirshfeld Difference C7       --C12      .       0.16 Ang.
PLAT234_ALERT_4_C Large Hirshfeld Difference C11      --C12      .       0.19 Ang.
PLAT234_ALERT_4_C Large Hirshfeld Difference C19      --C20      .       0.18 Ang.
PLAT241_ALERT_2_C High   'MainMol' Ueq as Compared to Neighbors of         N7 Check
PLAT241_ALERT_2_C High   'MainMol' Ueq as Compared to Neighbors of        C42 Check
PLAT242_ALERT_2_C Low    'MainMol' Ueq as Compared to Neighbors of         C8 Check
PLAT242_ALERT_2_C Low    'MainMol' Ueq as Compared to Neighbors of        C13 Check
PLAT242_ALERT_2_C Low    'MainMol' Ueq as Compared to Neighbors of        C21 Check
PLAT260_ALERT_2_C Large Average Ueq of Residue Including       O1      0.169 Check
PLAT341_ALERT_3_C Low Bond Precision on  C-C Bonds ...............    0.01027 Ang.
PLAT906_ALERT_3_C Large K Value in the Analysis of Variance ......      3.179 Check
PLAT911_ALERT_3_C Missing FCF Refl Between Thmin & STh/L=    0.595         73 Report
PLAT975_ALERT_2_C Check Calcd Resid. Dens.  1.02A   From N7            0.51 eA-3
PLAT978_ALERT_2_C Number C-C Bonds with Positive Residual Density.          0 Info
```

Alert level G

```
PLAT002_ALERT_2_G Number of Distance or Angle Restraints on AtSite          3 Note
PLAT003_ALERT_2_G Number of Uiso or Uij Restrained non-H Atoms ...         11 Report
PLAT007_ALERT_5_G Number of Unrefined Donor-H Atoms ..............          2 Report
PLAT042_ALERT_1_G Calc. and Reported MoietyFormula Strings  Differ     Please Check
PLAT045_ALERT_1_G Calculated and Reported Z Differ by a Factor ...       0.50 Check
PLAT066_ALERT_1_G Predicted and Reported Tmin&Tmax Range Identical          ? Check
PLAT072_ALERT_2_G SHELXL First  Parameter in WGHT  Unusually Large       0.11 Report
PLAT154_ALERT_1_G The s.u.'s on the Cell Angles are Equal ..(Note)      0.003 Degree
PLAT172_ALERT_4_G The CIF-Embedded .res File Contains DFIX Records          2 Report
PLAT177_ALERT_4_G The CIF-Embedded .res File Contains DELU Records          2 Report
```

```
PLAT199_ALERT_1_G Reported _cell_measurement_temperature ..... (K)        293 Check
PLAT200_ALERT_1_G Reported   _diffrn_ambient_temperature ..... (K)        293 Check
PLAT300_ALERT_4_G Atom Site Occupancy of O1         Constrained at        0.5 Check
PLAT300_ALERT_4_G Atom Site Occupancy of H101       Constrained at        0.5 Check
PLAT300_ALERT_4_G Atom Site Occupancy of H201       Constrained at        0.5 Check
PLAT302_ALERT_4_G Anion/Solvent/Minor-Residue Disorder (Resd  3  )       100% Note
PLAT304_ALERT_4_G Non-Integer Number of Atoms in ...... Resd  3          1.50 Check
PLAT794_ALERT_5_G Tentative Bond Valency for Fe1       (III)     .       3.29 Info
PLAT860_ALERT_3_G Number of Least-Squares Restraints .............         17 Note
PLAT883_ALERT_1_G No Info/Value for _atom_sites_solution_primary .     Please Do !
PLAT909_ALERT_3_G Percentage of I>2sig(I) Data at Theta(Max) Still        35% Note
PLAT933_ALERT_2_G Number of OMIT Records in Embedded .res File ...          5 Note
```

```
  0 ALERT level A = Most likely a serious problem - resolve or explain
  2 ALERT level B = A potentially serious problem, consider carefully
 23 ALERT level C = Check. Ensure it is not caused by an omission or oversight
 22 ALERT level G = General information/check it is not something unexpected

  7 ALERT type 1 CIF construction/syntax error, inconsistent or missing data
 20 ALERT type 2 Indicator that the structure model may be wrong or deficient
  5 ALERT type 3 Indicator that the structure quality may be low
 13 ALERT type 4 Improvement, methodology, query or suggestion
  2 ALERT type 5 Informative message, check
```

It is advisable to attempt to resolve as many as possible of the alerts in all categories. Often the minor alerts point to easily fixed oversights, errors and omissions in your CIF or refinement strategy, so attention to these fine details can be worthwhile. In order to resolve some of the more serious problems it may be necessary to carry out additional measurements or structure refinements. However, the purpose of your study may justify the reported deviations and the more serious of these should normally be commented upon in the discussion or experimental section of a paper or in the "special_details" fields of the CIF. checkCIF was carefully designed to identify outliers and unusual parameters, but every test has its limitations and alerts that are not important in a particular case may appear. Conversely, the absence of alerts does not guarantee there are no aspects of the results needing attention. It is up to the individual to critically assess their own results and, if necessary, seek expert advice.

Publication of your CIF in IUCr journals

A basic structural check has been run on your CIF. These basic checks will be run on all CIFs submitted for publication in IUCr journals (*Acta Crystallographica*, *Journal of Applied Crystallography*, *Journal of Synchrotron Radiation*); however, if you intend to submit to *Acta Crystallographica Section C* or *E* or *IUCrData*, you should make sure that full publication checks are run on the final version of your CIF prior to submission.

Publication of your CIF in other journals

Please refer to the *Notes for Authors* of the relevant journal for any special instructions relating to CIF submission.

PLATON version of 03/05/2019; check.def file version of 29/04/2019

Datablock 1 - ellipsoid plot

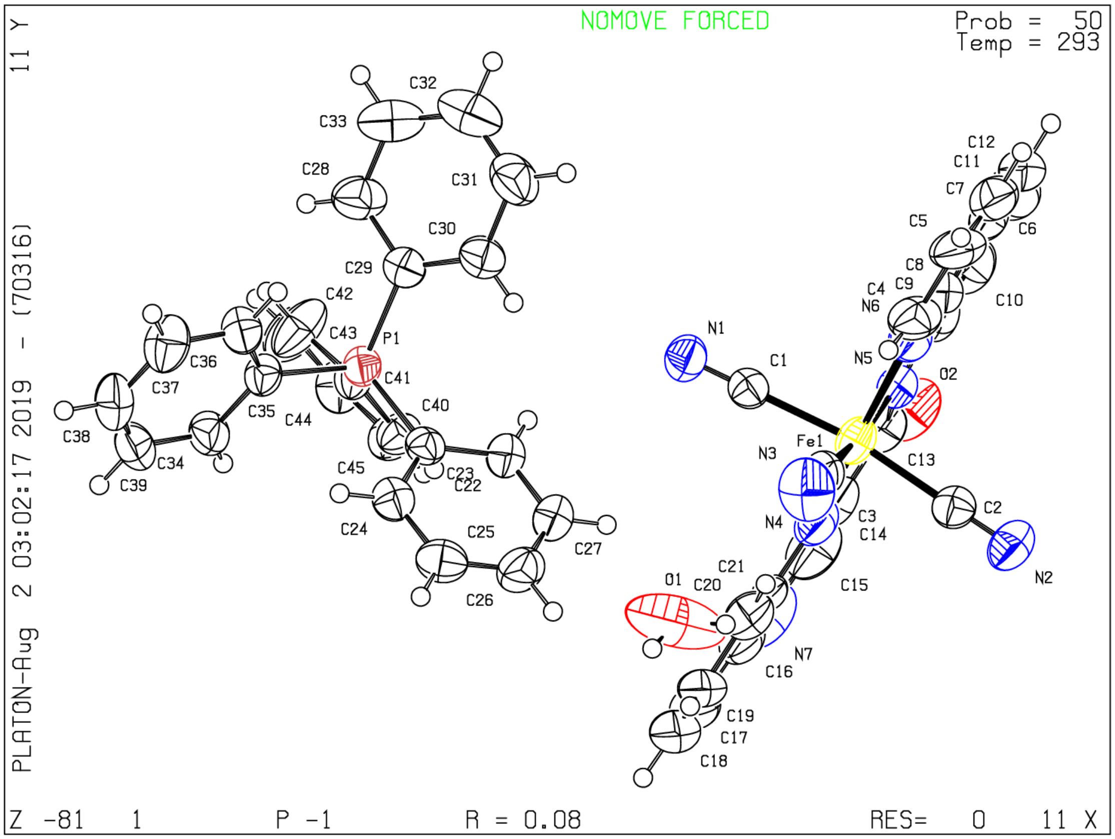

Supplement: Supplementary file 1 [file polymers-11-01585-s001.zip › checkcif 1.pdf]
