# Supplementary material for: Various Structural Types of Cyanide-Bridged FeIII–MnIII Bimetallic Coordination Polymers (CPs) and Polynuclear Clusters Based-on A New mer-Tricyanoiron(III)Building Block: Synthesis, Crystal Structures, and Magnetic Properties
Source: Polymers (Basel). 2019 Sep 27;11(10):1585. doi: 10.3390/polym11101585 (PMC6835830; doi:10.3390/polym11101585)

checkCIF/PLATON report

Structure factors have been supplied for datablock(s) 2

THIS REPORT IS FOR GUIDANCE ONLY. IF USED AS PART OF A REVIEW PROCEDURE FOR PUBLICATION, IT SHOULD NOT REPLACE THE EXPERTISE OF AN EXPERIENCED CRYSTALLOGRAPHIC REFEREE.

No syntax errors found. CIF dictionary Interpreting this report

Datablock: 2

```
Bond precision:  C-C = 0.0091 A          Wavelength=0.71073

Cell:           a=11.681(3)       b=14.778(4)       c=21.408(6)
                alpha=94.864(5)   beta=101.844(5)   gamma=90.613(5)
Temperature:    293 K
```

	Calculated	Reported
Volume	3602.3(17)	3602.3(16)
Space group	P -1	P -1
Hall group	-P 1	-P 1
Moiety formula	2(C39 H29 Br2 Fe Mn N9 O6), 2(C19 H22 Br2 Mn N2 O6), 2(Cl O4),	C39H29Br2FeMnN9O6,C19H22Br2MnN2O6,ClO4,1.5(Ch4O) 0.5H2O
Sum formula	C119 H116 Br8 Cl2 Fe2 Mn4 N22 O36	C59.50 H58 Br4 Cl Fe Mn2 N11 O18
Mr	3471.90	1735.99
Dx,g cm-3	1.600	1.600
Z	1	2
Mu (mm-1)	2.872	2.872
F000	1738.0	1738.0
F000’	1738.76	
h,k,lmax	13,17,25	13,17,25
Nref	12710	12538
Tmin,Tmax	0.427,0.517	0.471,0.558
Tmin’	0.395	

```
Correction method= # Reported T Limits: Tmin=0.471 Tmax=0.558
AbsCorr = MULTI-SCAN
```

```
Data completeness= 0.986          Theta(max)= 25.009

R(reflections)= 0.0514( 7569)     wR2(reflections)= 0.1595( 12538)

S = 1.008                   Npar= 890
```

The following ALERTS were generated. Each ALERT has the format
test-name_ALERT_alert-type_alert-level.
Click on the hyperlinks for more details of the test.

Alert level B

```
PLAT417_ALERT_2_B Short Inter D-H..H-D       H12A     ..H19B     .       2.06 Ang.
                                                  -1+x,y,z  =      1_455 Check
```

Author Response: This is maybe because of the intermolecular O-H...O and O-H...N hydrogen bond interactions.

Alert level C

```
PLAT220_ALERT_2_C Non-Solvent  Resd 1  C   Ueq(max)/Ueq(min) Range       3.4 Ratio
PLAT230_ALERT_2_C Hirshfeld Test Diff for    N7       --C14      .       5.7 s.u.
PLAT230_ALERT_2_C Hirshfeld Test Diff for    N7       --C16      .       5.5 s.u.
PLAT230_ALERT_2_C Hirshfeld Test Diff for    C7       --C8       .       5.9 s.u.
PLAT232_ALERT_2_C Hirshfeld Test Diff (M-X)  Mn1      --O6       .       6.5 s.u.
PLAT234_ALERT_4_C Large Hirshfeld Difference N5       --C21      .      0.17 Ang.
PLAT234_ALERT_4_C Large Hirshfeld Difference C15      --C16      .      0.17 Ang.
PLAT241_ALERT_2_C High   'MainMol' Ueq as Compared to Neighbors of        N7 Check
PLAT241_ALERT_2_C High   'MainMol' Ueq as Compared to Neighbors of       C18 Check
PLAT241_ALERT_2_C High   'MainMol' Ueq as Compared to Neighbors of       C55 Check
PLAT242_ALERT_2_C Low    'MainMol' Ueq as Compared to Neighbors of        O3 Check
PLAT242_ALERT_2_C Low    'MainMol' Ueq as Compared to Neighbors of       C15 Check
PLAT260_ALERT_2_C Large Average Ueq of Residue Including      Cl1     0.137 Check
PLAT260_ALERT_2_C Large Average Ueq of Residue Including      O17     0.148 Check
PLAT260_ALERT_2_C Large Average Ueq of Residue Including      O19     0.106 Check
PLAT260_ALERT_2_C Large Average Ueq of Residue Including      O18     0.134 Check
PLAT341_ALERT_3_C Low Bond Precision on  C-C Bonds ...............   0.00906 Ang.
PLAT360_ALERT_2_C Short  C(sp3)-C(sp3) Bond  C55      - C56      .      1.35 Ang.
PLAT369_ALERT_2_C Long   C(sp2)-C(sp2) Bond  C13      - C21      .      1.53 Ang.
PLAT410_ALERT_2_C Short Intra H...H Contact  H55A     ..H56B     .      1.96 Ang.
                                                  x,y,z  =      1_555 Check
PLAT410_ALERT_2_C Short Intra H...H Contact  H55B     ..H56A     .      1.96 Ang.
                                                  x,y,z  =      1_555 Check
PLAT601_ALERT_2_C Structure Contains Solvent Accessible VOIDS of .        39 Ang**3
PLAT905_ALERT_3_C Negative K value in the Analysis of Variance ...    -2.430 Report
PLAT911_ALERT_3_C Missing FCF Refl Between Thmin & STh/L=    0.595       169 Report
PLAT934_ALERT_3_C Number of (Iobs-Icalc)/SigmaW > 10 Outliers ....         1 Check
PLAT975_ALERT_2_C Check Calcd Resid. Dens.  0.59A   From O17          0.41 eA-3
PLAT978_ALERT_2_C Number C-C Bonds with Positive Residual Density.         0 Info
```

Alert level G

```
FORMU01_ALERT_1_G  There is a discrepancy between the atom counts in the
            _chemical_formula_sum and _chemical_formula_moiety. This is
            usually due to the moiety formula being in the wrong format.
            Atom count from _chemical_formula_sum:   C59.5 H58 Br4 Cl1 Fe1 Mn2 N11
```

```
            Atom count from _chemical_formula_moiety:C58.75 H54 Br4 Cl1 Fe1 Mn2 N1
PLAT002_ALERT_2_G Number of Distance or Angle Restraints on AtSite          4 Note
PLAT003_ALERT_2_G Number of Uiso or Uij Restrained non-H Atoms ...          3 Report
PLAT007_ALERT_5_G Number of Unrefined Donor-H Atoms .............           9 Report
PLAT042_ALERT_1_G Calc. and Reported MoietyFormula Strings  Differ     Please Check
PLAT045_ALERT_1_G Calculated and Reported Z Differ by a Factor ...       0.50 Check
PLAT154_ALERT_1_G The s.u.'s on the Cell Angles are Equal ..(Note)      0.005 Degree
PLAT172_ALERT_4_G The CIF-Embedded .res File Contains DFIX Records          2 Report
PLAT178_ALERT_4_G The CIF-Embedded .res File Contains SIMU Records          1 Report
PLAT199_ALERT_1_G Reported _cell_measurement_temperature ..... (K)        293 Check
PLAT200_ALERT_1_G Reported   _diffrn_ambient_temperature ..... (K)        293 Check
PLAT244_ALERT_4_G Low    'Solvent' Ueq as Compared to Neighbors of        Cl1 Check
PLAT300_ALERT_4_G Atom Site Occupancy of O17        Constrained at        0.5 Check
PLAT300_ALERT_4_G Atom Site Occupancy of C58        Constrained at        0.5 Check
PLAT300_ALERT_4_G Atom Site Occupancy of H17A       Constrained at        0.5 Check
PLAT300_ALERT_4_G Atom Site Occupancy of H58A       Constrained at        0.5 Check
PLAT300_ALERT_4_G Atom Site Occupancy of H58B       Constrained at        0.5 Check
PLAT300_ALERT_4_G Atom Site Occupancy of H58C       Constrained at        0.5 Check
PLAT300_ALERT_4_G Atom Site Occupancy of O18        Constrained at        0.5 Check
PLAT300_ALERT_4_G Atom Site Occupancy of H18A       Constrained at        0.5 Check
PLAT300_ALERT_4_G Atom Site Occupancy of H18B       Constrained at        0.5 Check
PLAT302_ALERT_4_G Anion/Solvent/Minor-Residue Disorder (Resd  4  )       100% Note
PLAT302_ALERT_4_G Anion/Solvent/Minor-Residue Disorder (Resd  6  )       100% Note
PLAT304_ALERT_4_G Non-Integer Number of Atoms in ...... Resd  6          1.50 Check
PLAT432_ALERT_2_G Short Inter X...Y Contact  O15      ..C46              2.99 Ang.
                                                      x,y,z  =      1_555 Check
PLAT779_ALERT_4_G Suspect or Irrelevant (Bond) Angle in CIF .... #         70 Check
              H17A -O17  -H17A    1.555   1.555   1.555             0.00 Deg.
PLAT779_ALERT_4_G Suspect or Irrelevant (Bond) Angle in CIF .... #         74 Check
              H18B -O18  -H18B    1.555   1.555   1.555             0.00 Deg.
PLAT779_ALERT_4_G Suspect or Irrelevant (Bond) Angle in CIF .... #         75 Check
              H18A -O18  -H18A    1.555   1.555   1.555             0.00 Deg.
PLAT794_ALERT_5_G Tentative Bond Valency for Fe1       (III)     .       3.42 Info
PLAT794_ALERT_5_G Tentative Bond Valency for Mn1       (I)       .       0.84 Info
PLAT794_ALERT_5_G Tentative Bond Valency for Mn2       (I)       .       0.86 Info
PLAT802_ALERT_4_G CIF Input Record(s) with more than 80 Characters          1 Info
PLAT860_ALERT_3_G Number of Least-Squares Restraints ............          20 Note
PLAT883_ALERT_1_G No Info/Value for _atom_sites_solution_primary .     Please Do !
PLAT910_ALERT_3_G Missing # of FCF Reflection(s) Below Theta(Min).          4 Note
PLAT933_ALERT_2_G Number of OMIT Records in Embedded .res File ...         36 Note
```

---

0 **ALERT level A** = Most likely a serious problem - resolve or explain
1 **ALERT level B** = A potentially serious problem, consider carefully
27 **ALERT level C** = Check. Ensure it is not caused by an omission or oversight
36 **ALERT level G** = General information/check it is not something unexpected

7 ALERT type 1 CIF construction/syntax error, inconsistent or missing data
26 ALERT type 2 Indicator that the structure model may be wrong or deficient
6 ALERT type 3 Indicator that the structure quality may be low
21 ALERT type 4 Improvement, methodology, query or suggestion
4 ALERT type 5 Informative message, check

It is advisable to attempt to resolve as many as possible of the alerts in all categories. Often the minor alerts point to easily fixed oversights, errors and omissions in your CIF or refinement strategy, so attention to these fine details can be worthwhile. In order to resolve some of the more serious problems it may be necessary to carry out additional measurements or structure refinements. However, the purpose of your study may justify the reported deviations and the more serious of these should normally be commented upon in the discussion or experimental section of a paper or in the "special_details" fields of the CIF. checkCIF was carefully designed to identify outliers and unusual parameters, but every test has its limitations and alerts that are not important in a particular case may appear. Conversely, the absence of alerts does not guarantee there are no aspects of the results needing attention. It is up to the individual to critically assess their own results and, if necessary, seek expert advice.

Publication of your CIF in IUCr journals

A basic structural check has been run on your CIF. These basic checks will be run on all CIFs submitted for publication in IUCr journals (*Acta Crystallographica*, *Journal of Applied Crystallography*, *Journal of Synchrotron Radiation*); however, if you intend to submit to *Acta Crystallographica Section C* or *E* or *IUCrData*, you should make sure that full publication checks are run on the final version of your CIF prior to submission.

Publication of your CIF in other journals

Please refer to the *Notes for Authors* of the relevant journal for any special instructions relating to CIF submission.

PLATON version of 03/05/2019; check.def file version of 29/04/2019

Datablock 2 - ellipsoid plot

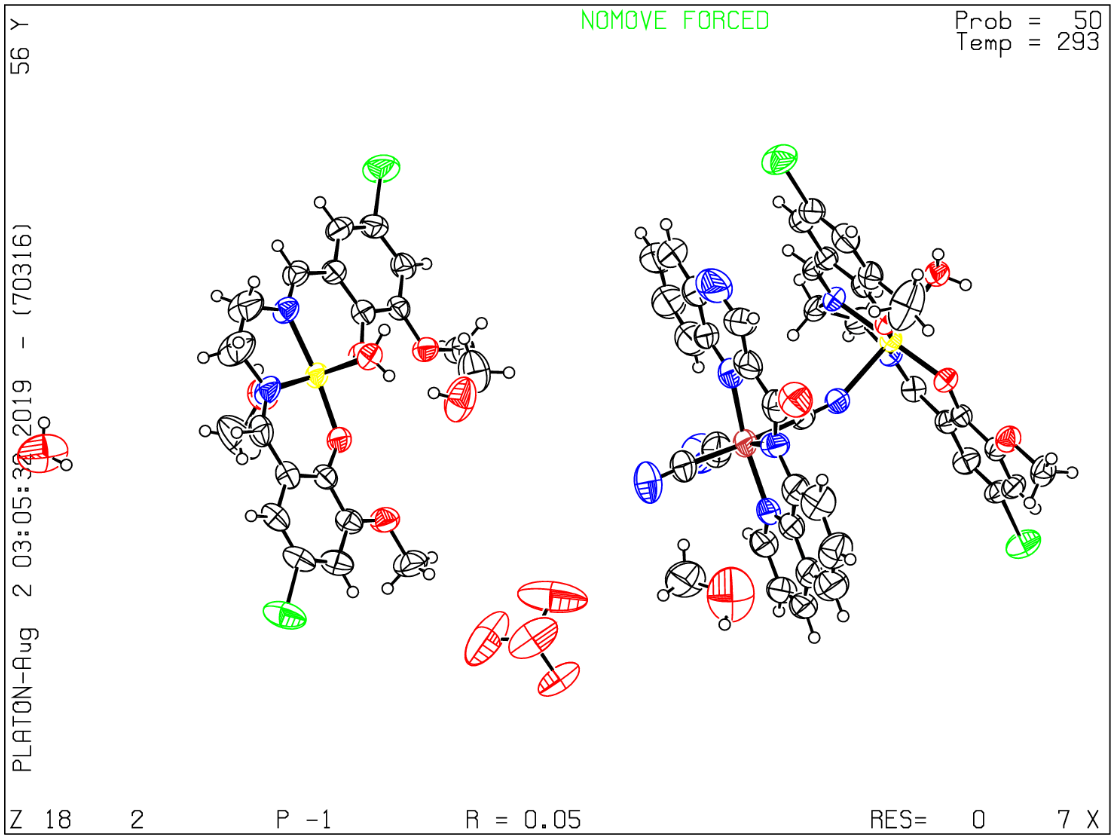

Supplement: Supplementary file 1 [file polymers-11-01585-s001.zip › checkcif 2.pdf]
