# Supplementary material for: Various Structural Types of Cyanide-Bridged FeIII–MnIII Bimetallic Coordination Polymers (CPs) and Polynuclear Clusters Based-on A New mer-Tricyanoiron(III)Building Block: Synthesis, Crystal Structures, and Magnetic Properties
Source: Polymers (Basel). 2019 Sep 27;11(10):1585. doi: 10.3390/polym11101585 (PMC6835830; doi:10.3390/polym11101585)

checkCIF/PLATON report

Structure factors have been supplied for datablock(s) test

THIS REPORT IS FOR GUIDANCE ONLY. IF USED AS PART OF A REVIEW PROCEDURE FOR PUBLICATION, IT SHOULD NOT REPLACE THE EXPERTISE OF AN EXPERIENCED CRYSTALLOGRAPHIC REFEREE.

No syntax errors found. CIF dictionary Interpreting this report

Datablock: test

Bond precision: C-C = 0.0101 A Wavelength=0.71073

Cell:	a=14.3852(11)	b=15.3085(11)	c=21.6557(17)
	alpha=90	beta=105.645(1)	gamma=90
Temperature:	293 K		

	Calculated	Reported
Volume	4592.2(6)	4592.2(6)
Space group	P 21/n	P 21/n
Hall group	-P 2yn	-P 2yn
Moiety formula	C41 H33 Fe Mn N9 O3, C H1.50 N0.50, C0.50 H2 O0.50, C H4 O, H0.	?
Sum formula	C43.50 H41 Fe Mn N9.50 O4.75	C43.50 H41 Fe Mn N9.50 O4.75
Mr	883.65	883.65
Dx,g cm-3	1.278	1.278
Z	4	4
Mu (mm-1)	0.643	0.643
F000	1830.0	1830.0
F000’	1833.32	
h,k,lmax	17,18,25	17,18,25
Nref	8096	8062
Tmin,Tmax	0.837,0.891	0.825,0.894
Tmin’	0.819	

Correction method= # Reported T Limits: Tmin=0.825 Tmax=0.894
AbsCorr = MULTI-SCAN

Data completeness= 0.996 Theta(max)= 25.009

R(reflections)= 0.0777(6079) wR2(reflections)= 0.2482(8062)

S = 1.041 Npar= 577

The following ALERTS were generated. Each ALERT has the format
test-name_ALERT_alert-type_alert-level.
Click on the hyperlinks for more details of the test.

Alert level C

```
DIFMX02_ALERT_1_C  The maximum difference density is > 0.1*ZMAX*0.75
            The relevant atom site should be identified.
PLAT094_ALERT_2_C Ratio of Maximum / Minimum Residual Density ....       2.35 Report
PLAT097_ALERT_2_C Large Reported Max.  (Positive) Residual Density       2.12 eA-3
PLAT220_ALERT_2_C Non-Solvent  Resd 1  C   Ueq(max)/Ueq(min) Range        3.1 Ratio
PLAT234_ALERT_4_C Large Hirshfeld Difference N7       --C11      .       0.17 Ang.
PLAT241_ALERT_2_C High   'MainMol' Ueq as Compared to Neighbors of         N7 Check
PLAT241_ALERT_2_C High   'MainMol' Ueq as Compared to Neighbors of        C29 Check
PLAT241_ALERT_2_C High   'MainMol' Ueq as Compared to Neighbors of        C34 Check
PLAT242_ALERT_2_C Low    'MainMol' Ueq as Compared to Neighbors of         C9 Check
PLAT242_ALERT_2_C Low    'MainMol' Ueq as Compared to Neighbors of        C11 Check
PLAT260_ALERT_2_C Large Average Ueq of Residue Including       N10      0.106 Check
PLAT260_ALERT_2_C Large Average Ueq of Residue Including        O6      0.121 Check
PLAT260_ALERT_2_C Large Average Ueq of Residue Including        O5      0.134 Check
PLAT341_ALERT_3_C Low Bond Precision on  C-C Bonds ...............    0.01006 Ang.
PLAT911_ALERT_3_C Missing FCF Refl Between Thmin & STh/L=    0.595         32 Report
PLAT918_ALERT_3_C Reflection(s) with I(obs) much Smaller I(calc) .          2 Check
PLAT934_ALERT_3_C Number of (Iobs-Icalc)/SigmaW > 10 Outliers ....          1 Check
PLAT971_ALERT_2_C Check Calcd Resid. Dens.  0.82A   From Fe1             2.07 eA-3
PLAT975_ALERT_2_C Check Calcd Resid. Dens.  0.94A   From O5              1.23 eA-3
PLAT975_ALERT_2_C Check Calcd Resid. Dens.  0.61A   From O5              1.04 eA-3
PLAT975_ALERT_2_C Check Calcd Resid. Dens.  0.67A   From O5              1.04 eA-3
PLAT975_ALERT_2_C Check Calcd Resid. Dens.  1.06A   From O5              0.89 eA-3
PLAT975_ALERT_2_C Check Calcd Resid. Dens.  1.05A   From N7              0.51 eA-3
```

Alert level G

```
PLAT003_ALERT_2_G Number of Uiso or Uij Restrained non-H Atoms ...          8 Report
PLAT004_ALERT_5_G Polymeric Structure Found with Maximum Dimension          1 Info
PLAT007_ALERT_5_G Number of Unrefined Donor-H Atoms ..............          4 Report
PLAT066_ALERT_1_G Predicted and Reported Tmin&Tmax Range Identical          ? Check
PLAT072_ALERT_2_G SHELXL First  Parameter in WGHT  Unusually Large       0.14 Report
PLAT083_ALERT_2_G SHELXL Second Parameter in WGHT  Unusually Large       9.83 Why ?
PLAT177_ALERT_4_G The CIF-Embedded .res File Contains DELU Records          1 Report
PLAT186_ALERT_4_G The CIF-Embedded .res File Contains ISOR Records          1 Report
PLAT199_ALERT_1_G Reported _cell_measurement_temperature ..... (K)        293 Check
PLAT200_ALERT_1_G Reported   _diffrn_ambient_temperature ..... (K)        293 Check
PLAT300_ALERT_4_G Atom Site Occupancy of N10        Constrained at        0.5 Check
PLAT300_ALERT_4_G Atom Site Occupancy of C45        Constrained at        0.5 Check
PLAT300_ALERT_4_G Atom Site Occupancy of C46        Constrained at        0.5 Check
PLAT300_ALERT_4_G Atom Site Occupancy of H47A       Constrained at        0.5 Check
PLAT300_ALERT_4_G Atom Site Occupancy of H47B       Constrained at        0.5 Check
PLAT300_ALERT_4_G Atom Site Occupancy of H47C       Constrained at        0.5 Check
PLAT300_ALERT_4_G Atom Site Occupancy of O4         Constrained at        0.5 Check
PLAT300_ALERT_4_G Atom Site Occupancy of C43        Constrained at        0.5 Check
PLAT300_ALERT_4_G Atom Site Occupancy of H4         Constrained at        0.5 Check
PLAT300_ALERT_4_G Atom Site Occupancy of H43A       Constrained at        0.5 Check
PLAT300_ALERT_4_G Atom Site Occupancy of H43B       Constrained at        0.5 Check
PLAT300_ALERT_4_G Atom Site Occupancy of H43C       Constrained at        0.5 Check
PLAT300_ALERT_4_G Atom Site Occupancy of O5         Constrained at       0.25 Check
PLAT300_ALERT_4_G Atom Site Occupancy of H5B        Constrained at       0.25 Check
PLAT300_ALERT_4_G Atom Site Occupancy of H5C        Constrained at       0.25 Check
PLAT302_ALERT_4_G Anion/Solvent/Minor-Residue Disorder (Resd  2  )       100% Note
PLAT302_ALERT_4_G Anion/Solvent/Minor-Residue Disorder (Resd  3  )       100% Note
```

```
PLAT302_ALERT_4_G Anion/Solvent/Minor-Residue Disorder (Resd  5  )       100% Note
PLAT335_ALERT_2_G Check Large C6 Ring C-C Range C4       -C9             0.17 Ang.
PLAT794_ALERT_5_G Tentative Bond Valency for Fe1       (III)     .       3.31 Info
PLAT794_ALERT_5_G Tentative Bond Valency for Mn1       (I)       .       0.82 Info
PLAT860_ALERT_3_G Number of Least-Squares Restraints .............         21 Note
PLAT883_ALERT_1_G No Info/Value for _atom_sites_solution_primary .     Please Do !
PLAT909_ALERT_3_G Percentage of I>2sig(I) Data at Theta(Max) Still        49% Note
PLAT910_ALERT_3_G Missing # of FCF Reflection(s) Below Theta(Min).          2 Note
PLAT913_ALERT_3_G Missing # of Very Strong Reflections in FCF ....          2 Note
PLAT933_ALERT_2_G Number of OMIT Records in Embedded .res File ...         30 Note
PLAT978_ALERT_2_G Number C-C Bonds with Positive Residual Density.          1 Info
```

```
   0 ALERT level A = Most likely a serious problem - resolve or explain
   0 ALERT level B = A potentially serious problem, consider carefully
  23 ALERT level C = Check. Ensure it is not caused by an omission or oversight
  38 ALERT level G = General information/check it is not something unexpected

   5 ALERT type 1 CIF construction/syntax error, inconsistent or missing data
  23 ALERT type 2 Indicator that the structure model may be wrong or deficient
   8 ALERT type 3 Indicator that the structure quality may be low
  21 ALERT type 4 Improvement, methodology, query or suggestion
   4 ALERT type 5 Informative message, check
```

It is advisable to attempt to resolve as many as possible of the alerts in all categories. Often the minor alerts point to easily fixed oversights, errors and omissions in your CIF or refinement strategy, so attention to these fine details can be worthwhile. In order to resolve some of the more serious problems it may be necessary to carry out additional measurements or structure refinements. However, the purpose of your study may justify the reported deviations and the more serious of these should normally be commented upon in the discussion or experimental section of a paper or in the "special_details" fields of the CIF. checkCIF was carefully designed to identify outliers and unusual parameters, but every test has its limitations and alerts that are not important in a particular case may appear. Conversely, the absence of alerts does not guarantee there are no aspects of the results needing attention. It is up to the individual to critically assess their own results and, if necessary, seek expert advice.

Publication of your CIF in IUCr journals

A basic structural check has been run on your CIF. These basic checks will be run on all CIFs submitted for publication in IUCr journals (*Acta Crystallographica*, *Journal of Applied Crystallography*, *Journal of Synchrotron Radiation*); however, if you intend to submit to *Acta Crystallographica Section C* or *E* or *IUCrData*, you should make sure that full publication checks are run on the final version of your CIF prior to submission.

Publication of your CIF in other journals

Please refer to the *Notes for Authors* of the relevant journal for any special instructions relating to CIF submission.

PLATON version of 03/05/2019; check.def file version of 29/04/2019

Datablock test - ellipsoid plot

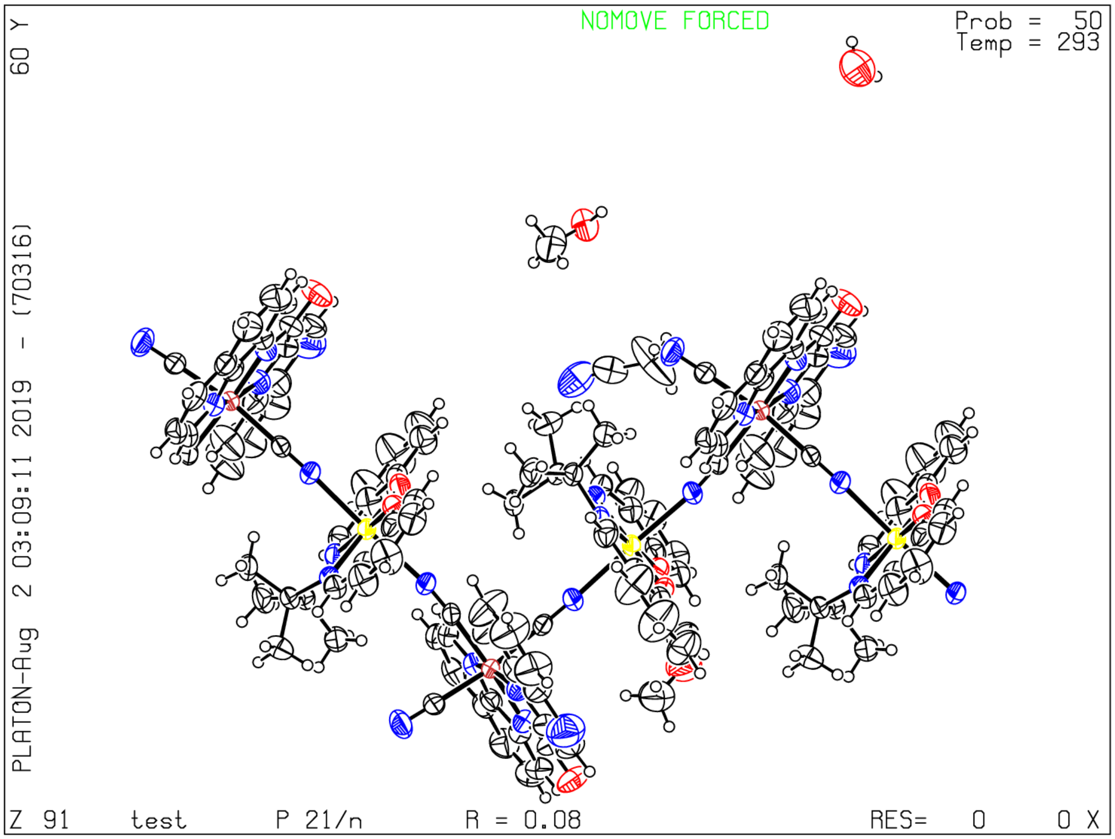

Supplement: Supplementary file 1 [file polymers-11-01585-s001.zip › checkcif 5.pdf]
