# Supplementary material for: Various Structural Types of Cyanide-Bridged FeIII–MnIII Bimetallic Coordination Polymers (CPs) and Polynuclear Clusters Based-on A New mer-Tricyanoiron(III)Building Block: Synthesis, Crystal Structures, and Magnetic Properties
Source: Polymers (Basel). 2019 Sep 27;11(10):1585. doi: 10.3390/polym11101585 (PMC6835830; doi:10.3390/polym11101585)
Supplement: Supplementary file 1 [file polymers-11-01585-s001.zip › Supporting Information 2019-9-20.docx]

**Supporting Information**

Various structural types of cyanide-bridged Fe^III^-Mn^III^ bimetallic coordination polymers(CPs) and polynuclear clusters based-on a new *mer-*tricyanoiron(III) building block:synthesis, crystal structures and magnetic properties

Wenlong Lan^1^, Xiaoyun Hao^1^, Yong Dou^1^, Zhen zhou^1^, LuYang^1^, Hui Liu^1^,Dacheng Li^2^, Yunhui Dong^1^, Lingqian Kong^3^ and Daopeng Zhang^1,^*

| **Table S1** Details of the Crystal Parameters, Data Collection, and Refinement for Complexes **1-5** | | | | | |
| --- | --- | --- | --- | --- | --- |
|  | **1** | **2** | **3** | **4** | **5** |
| Formula | C_45_H_32_Fe  N_7_O_1.5_P | C_59.5_H_58_Br_4_Cl  FeMn_2_N_11_O_18_ | C_41_H_35.5_Fe  MnN_9_O_6.25_ | C_33_H_29_Fe  MnN_9_O_3_ | C_43.5_H_41_Fe  MnN_9.5_O_4.75_ |
| Formula  weight | 781.59 | 1735.99 | 865.07 | 710.44 | 883.65 |
| Crystal  system | Triclinic | Triclinic | Triclinic | Monoclinic | Monoclinic |
| Space  group | *P-1* | *P-1* | *P-1* | *P2(1)/n* | *P2(1)/n* |
| *a*/A˚ | 9.3316(16) | 11.681(3) | 10.009(7) | 8.886(3) | 14.3852(11) |
| *b*/A˚ | 14.805(3) | 14.778(4) | 13.855(10) | 19.845(6) | 15.3085(11) |
| *c*/A˚ | 15.010(3) | 21.408(6) | 32.09(2) | 19.662(5) | 21.6557(17) |
| *α*/deg | 109.812(3) | 94.864(5) | 77.747(14) | 90 | 90 |
| *β*/deg | 97.307(3) | 101.844(5) | 88.594(18) | 99.847(6) | 105.645(10) |
| *γ*/deg | 99.965(3) | 90.613(5) | 87.637(13) | 90 | 90 |
| V/Å³ | 1882.5(6) | 3602.3(16) | 4345(6) | 3416.0(17) | 4592.2(6) |
| Z | 2 | 2 | 4 | 4 | 4 |
| F(000) | 808 | 1738 | 3564 | 1460 | 1830 |
| Reﬂections collected/  unique(Rint) | 9273/6567 (0.0292) | 17872/12538 (0.0224) | 21302/14905(0.0579) | 14269/4907 (0.1112) | 22457/8062 (0.0325) |
| Goodness-of-ﬁt | 1.029 | 1.008 | 0.987 | 1.015 | 1.041 |
| R_1_[I >2σ(I)] | 0.0777 | 0.0514 | 0.0909 | 0.0960 | 0.0777 |
| wR_2_[I >2σ(I)] | 0.1957 | 0.1346 | 0.2314 | 0.2193 | 0.2253 |

**Table S2** Selected bond lengths (Å) and angles (deg) for **1**-**3**.

| **1** |  | **2** |  | **3** |  |
| --- | --- | --- | --- | --- | --- |
| Fe1-C1 | 1.958(6) | Fe1-C1 | 1.949(6) | Fe1-C1 | 1.962(9) |
| Fe1-C2 | 1.968(6) | Fe1-C2 | 1.944(6) | Fe1-C2 | 1.961(10) |
| Fe1-C3 | 1.931(6) | Fe1-C3 | 1.940(9) | Fe1-C3 | 1.981(10) |
| Fe1-N4 | 2.004(5) | Fe1-N4 | 1.991(5) | Fe1-N4 | 2.052(8) |
| Fe1-N5 | 1.945(5) | Fe1-N5 | 1.906(5) | Fe1-N5 | 1.945(8) |
| Fe1-N6 | 2.060(5) | Fe1-N6 | 2.066(5) | Fe1-N6 | 2.013(7) |
| Fe1-C1-N1 | 178.7(5) | Mn1-N1 | 2.253(5) | Mn1-N1 | 2.248(6) |
| Fe1-C2-N2 | 179.2(5) | Mn1-N8 | 1.990(4) | Mn1-N8 | 1.999(7) |
| Fe1-C3-N3 | 176.4(6) | Mn1-N9 | 1.990(4) | Mn1-N9 | 1.973(8) |
|  |  | Mn1-O2 | 1.877(3) | Mn1-O1 | 1.895(5) |
|  |  | Mn1-O4 | 1.887(3) | Mn1-O3 | 1.871(5) |
|  |  | Mn1-O6 | 2.308(3) | Mn1-O5 | 2.296(5) |
|  |  | Fe1-C1-N1 | 175.6(5) | Fe1-C1-N1 | 175.2(8) |
|  |  | Fe1-C2-N2 | 176.0(6) | Fe1-C2-N2 | 177.7(8) |
|  |  | Fe1-C3-N3 | 179.0(8) | Fe1-C3-N3 | 177.1(8) |
|  |  | Mn1-N1-C1 | 153.4(4) | Mn1-N1-C1 | 160.2(7) |
|  |  |  |  | Mn(2)-O(10)-C(43) | 164.3(7) |

**Table S3** Selected bond lengths (Å) and angles (deg) for **4**-**5**.

| **4** |  | **5** |  |
| --- | --- | --- | --- |
| Fe1-C1 | 1.956(11) | Fe1-C1 | 1.967(5) |
| Fe1-C2 | 1.933(11) | Fe1-C2 | 1.958(6) |
| Fe1-C3 | 1.870(19) | Fe1-C3 | 1.966(5) |
| Fe1-N4 | 1.994(12) | Fe1-N4 | 2.011(5) |
| Fe1-N5 | 2.007(12) | Fe1-N5 | 1.916(5) |
| Fe1-N6 | 2.084(11) | Fe1-N6 | 2.047(5) |
| Mn1-N1 | 2.284(9) | Mn1-N1 | 2.315(4) |
| Mn1-N2 | 2.308(10) | Mn1-N3 | 2.326(4) |
| Mn1-N8 | 1.967(10) | Mn1-N8 | 1.985(4) |
| Mn1-N9 | 1.982(11) | Mn1-N9 | 1.982(4) |
| Mn1-O1 | 1.902(8) | Mn1-O1 | 1.896(3) |
| Mn1-O2 | 1.881(8) | Mn1-O2 | 1.874(4) |
| Fe1-C1-N1 | 174.6(10) | Fe1-C1-N1 | 177.1(5) |
| Fe1-C2-N2#2 | 177.6(10) | Fe1-C2-N2 | 178.2(5) |
| Fe1-C3-N3 | 174.2(16) | Fe1-C3-N3#2 | 172.3(4) |
| Mn1-N1-C1 | 154.8(9) | Mn1-N1-C1 | 173.4(4) |
| Mn1-N2-C2#1 | 159.0(9) | Mn1-N3-C3#1 | 172.4(4) |


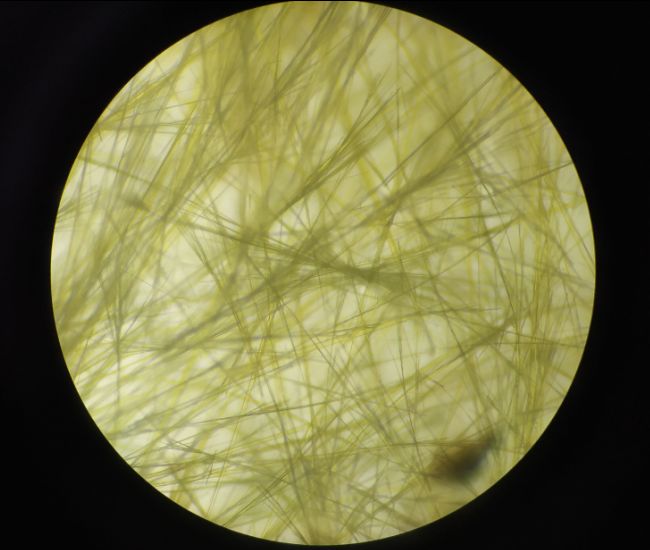


Figure S1. The image for the microcrystalline yellow color Hqcxq ligand under the microscope.


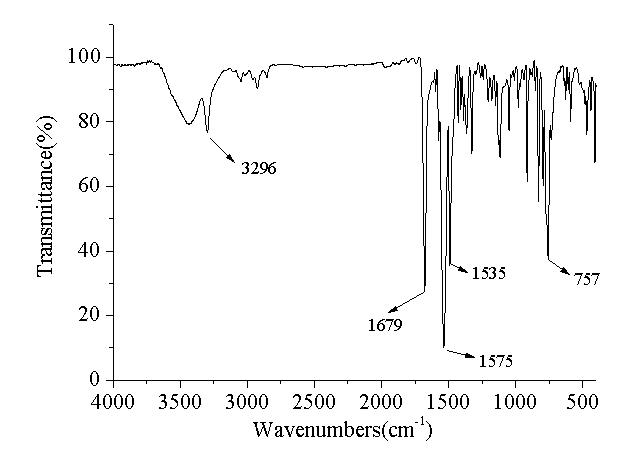


Figure S2. The IR of the Hqcxq ligand.


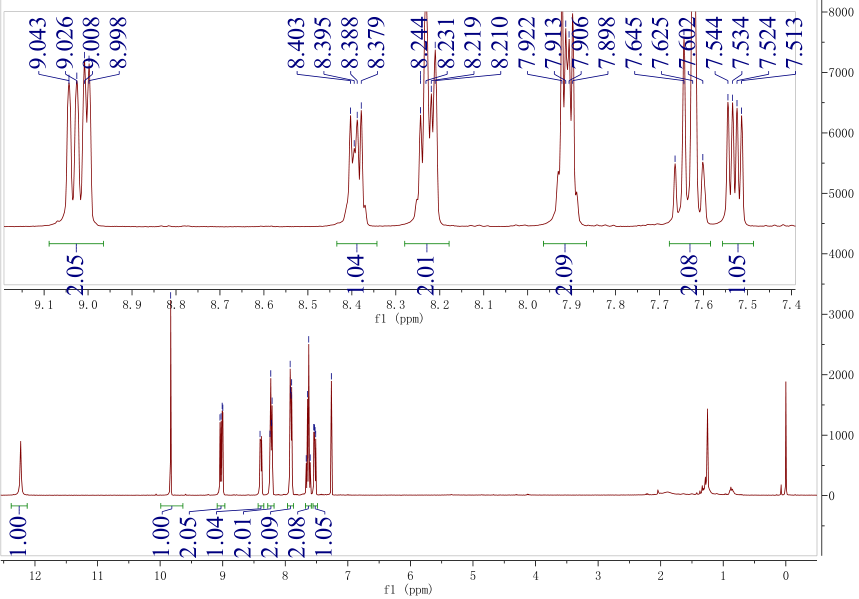

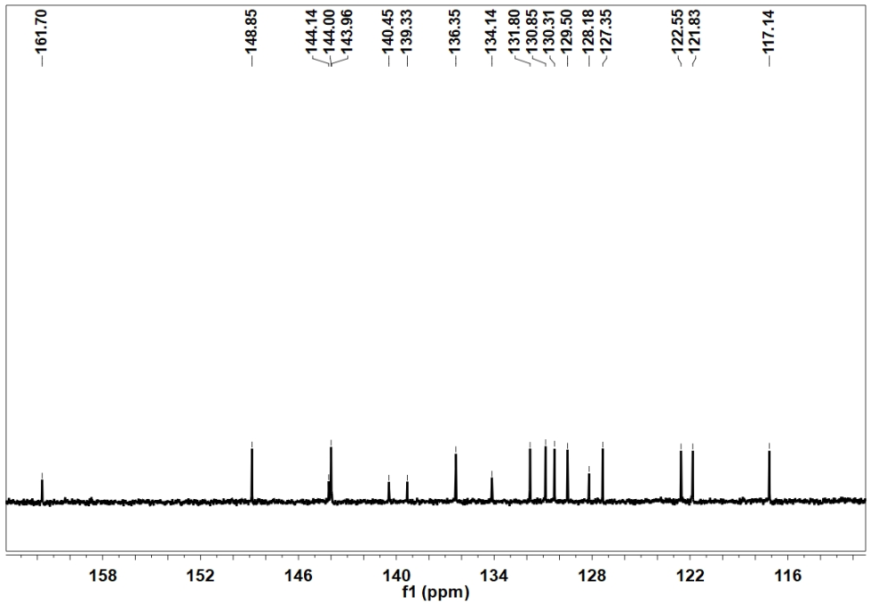


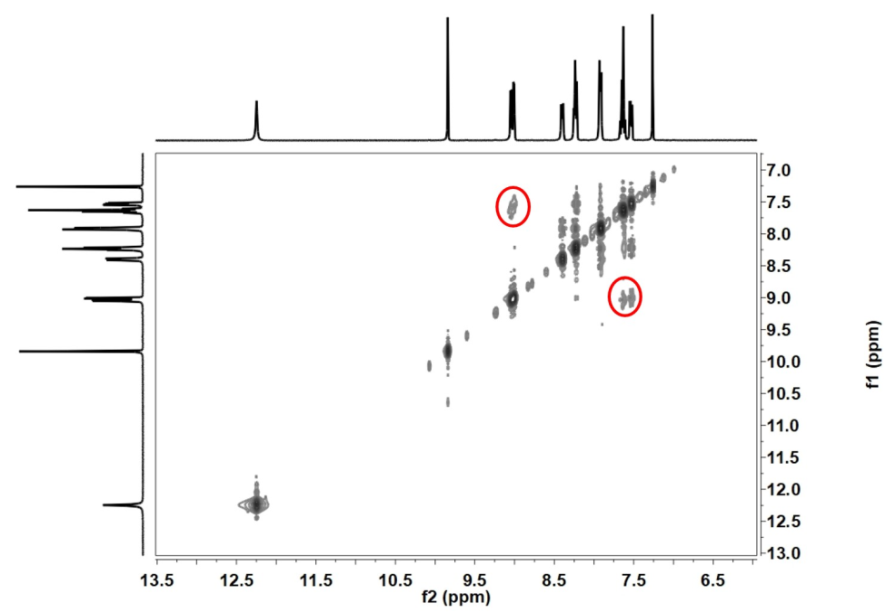


Figure S3. The ^1^H (top), ^13^C (middle) and 2D (bottom) NMR of the Hqxcq ligand.


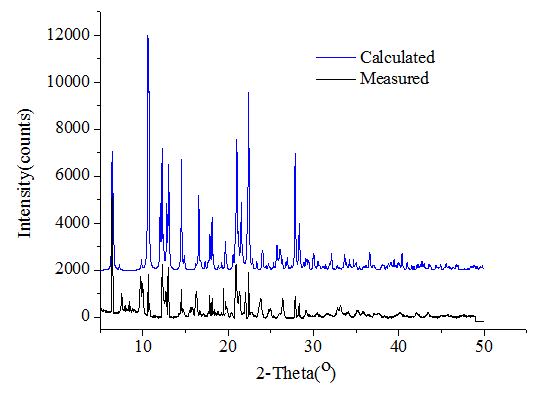


Figure S4. The calculated and measured XRD for the complex **1**.


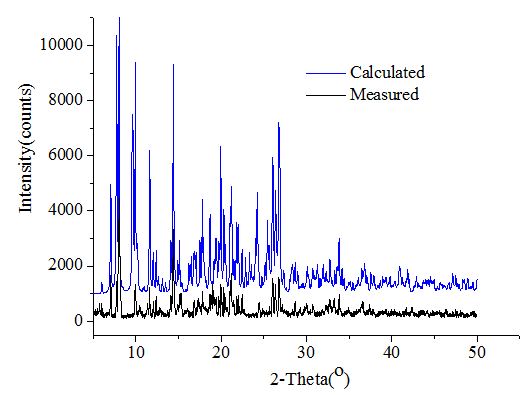


Figure S5.The calculated and measured XRD for the complex **2**.


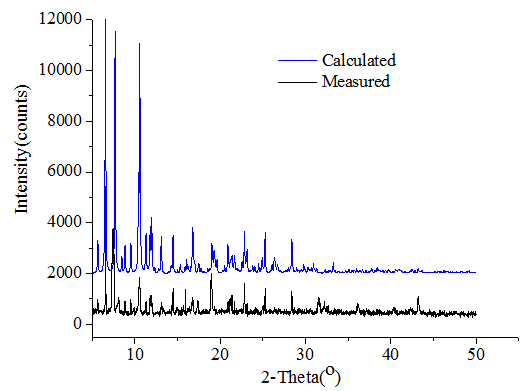


Figure S6.The calculated and measured XRD for the complex **3**.


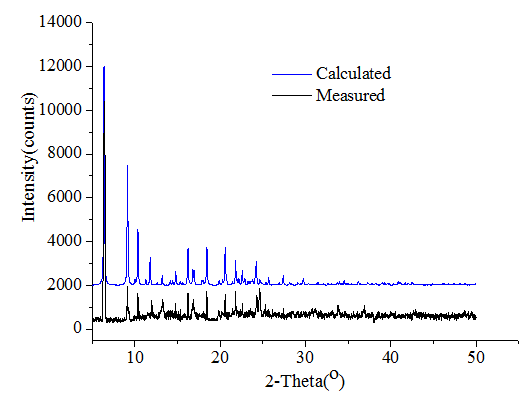


Figure S7.The calculated and measured XRD for the complex **4**.


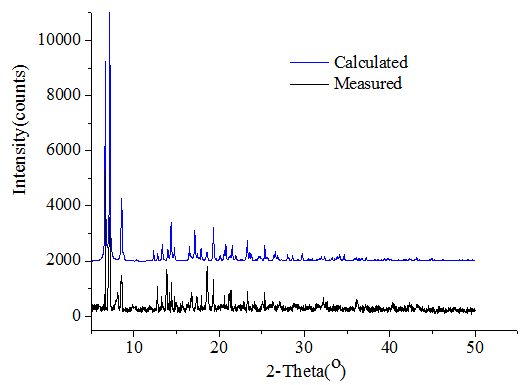


Figure S8.The calculated and measured XRD for the complex **5**.

Scheme S1.The synthesis scheme for the organic ligand and the complexes **1**-**5**.


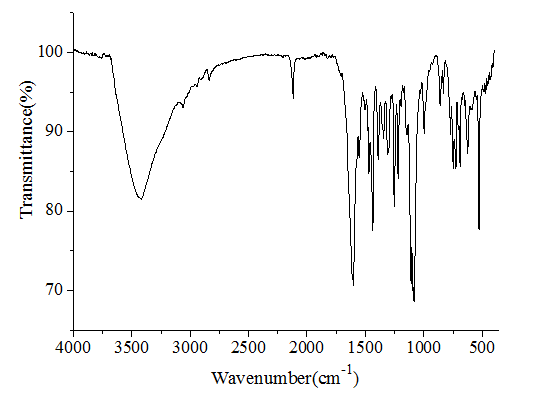


Figure S9. The IR of the complex **1**.


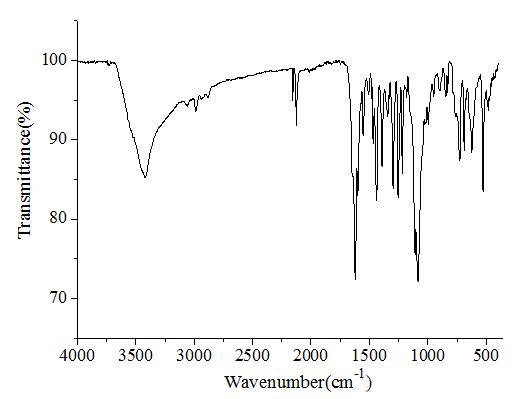


Figure S10. The IR of the complex **2**.


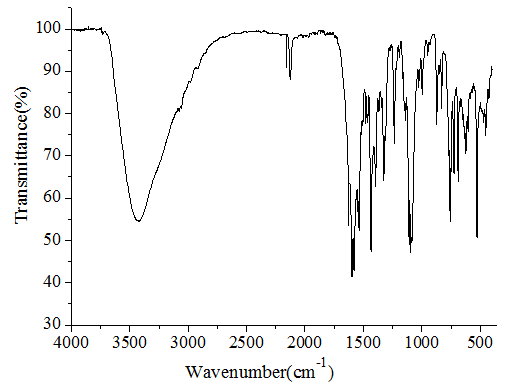


Figure S11. The IR of the complex **3**.


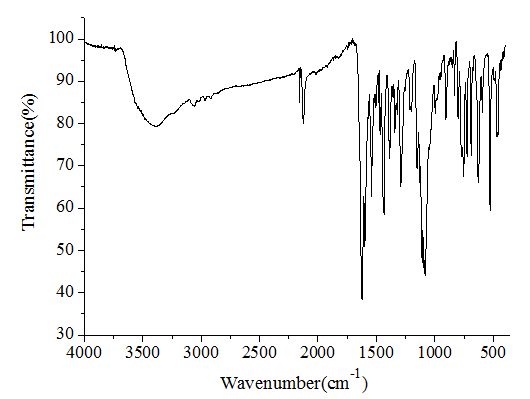


Figure S12. The IR of the complex **4**.


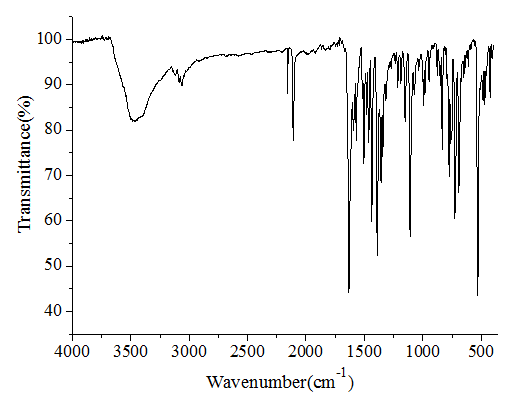


Figure S13. The IR of the complex **5**.
