# Supplementary material for: Various Structural Types of Cyanide-Bridged FeIII–MnIII Bimetallic Coordination Polymers (CPs) and Polynuclear Clusters Based-on A New mer-Tricyanoiron(III)Building Block: Synthesis, Crystal Structures, and Magnetic Properties
Source: Polymers (Basel). 2019 Sep 27;11(10):1585. doi: 10.3390/polym11101585 (PMC6835830; doi:10.3390/polym11101585)

checkCIF/PLATON report

Structure factors have been supplied for datablock(s) 3

THIS REPORT IS FOR GUIDANCE ONLY. IF USED AS PART OF A REVIEW PROCEDURE FOR PUBLICATION, IT SHOULD NOT REPLACE THE EXPERTISE OF AN EXPERIENCED CRYSTALLOGRAPHIC REFEREE.

No syntax errors found. CIF dictionary Interpreting this report

Datablock: 3

```
Bond precision:  C-C = 0.0151 A          Wavelength=0.71073

Cell:           a=10.009(7)        b=13.855(10)      c=32.09(2)
                alpha=77.747(14)   beta=88.594(18)   gamma=87.637(13)
Temperature:    293 K
```

	Calculated	Reported
Volume	4344(5)	4345(6)
Space group	P -1	P -1
Hall group	-P 1	-P 1
Moiety formula	4(C41 H35 Fe Mn N9 O6), H2 O	?
Sum formula	C164 H142 Fe4 Mn4 N36 O25	C41 H35.50 Fe Mn N9 O6.25
Mr	3460.30	865.07
Dx,g cm-3	1.323	1.322
Z	1	4
Mu (mm-1)	0.680	0.680
F000	1782.0	1782.0
F000’	1785.35	
h,k,lmax	11,16,37	11,16,37
Nref	15138	14905
Tmin,Tmax	0.915,0.941	0.917,0.942
Tmin’	0.915	

```
Correction method= # Reported T Limits: Tmin=0.917 Tmax=0.942
AbsCorr = MULTI-SCAN

Data completeness= 0.985          Theta(max)= 24.899

R(reflections)= 0.0909( 6853)     wR2(reflections)= 0.2769( 14905)

S = 0.987                  Npar= 1058
```

```
The following ALERTS were generated. Each ALERT has the format
       test-name_ALERT_alert-type_alert-level.
Click on the hyperlinks for more details of the test.
```

Alert level A

```
PLAT602_ALERT_2_A VERY LARGE Solvent Accessible VOID(S) in Structure        ! Info
```

Alert level B

```
PLAT341_ALERT_3_B Low Bond Precision on  C-C Bonds ...............    0.01506 Ang.
PLAT420_ALERT_2_B D-H Without Acceptor       O13      --H213     .     Please Check
```

Author Response: The electronic density arround the disoredered solvent oxygen atom is very weak, making it difficulty to accurately confirm the direction and the position of the H atom bonded.

Alert level C

```
PLAT026_ALERT_3_C Ratio Observed / Unique Reflections (too) Low ..        46% Check
PLAT084_ALERT_3_C High wR2 Value (i.e. > 0.25) ...................       0.28 Report
PLAT230_ALERT_2_C Hirshfeld Test Diff for    N7       --C14      .        5.5 s.u.
PLAT230_ALERT_2_C Hirshfeld Test Diff for    C7       --C12      .        6.3 s.u.
PLAT230_ALERT_2_C Hirshfeld Test Diff for    C49      --C54      .        5.3 s.u.
PLAT234_ALERT_4_C Large Hirshfeld Difference N6       --C13      .       0.18 Ang.
PLAT234_ALERT_4_C Large Hirshfeld Difference N6       --C15      .       0.20 Ang.
PLAT234_ALERT_4_C Large Hirshfeld Difference N7       --C16      .       0.16 Ang.
PLAT234_ALERT_4_C Large Hirshfeld Difference C6       --C7       .       0.18 Ang.
PLAT234_ALERT_4_C Large Hirshfeld Difference C7       --C8       .       0.19 Ang.
PLAT234_ALERT_4_C Large Hirshfeld Difference C8       --C9       .       0.18 Ang.
PLAT234_ALERT_4_C Large Hirshfeld Difference C13      --C14      .       0.16 Ang.
PLAT234_ALERT_4_C Large Hirshfeld Difference C13      --C21      .       0.19 Ang.
PLAT234_ALERT_4_C Large Hirshfeld Difference C15      --C16      .       0.17 Ang.
PLAT234_ALERT_4_C Large Hirshfeld Difference C19      --C20      .       0.16 Ang.
PLAT234_ALERT_4_C Large Hirshfeld Difference C22      --C27      .       0.18 Ang.
PLAT234_ALERT_4_C Large Hirshfeld Difference C30      --C31      .       0.16 Ang.
PLAT234_ALERT_4_C Large Hirshfeld Difference C35      --C36      .       0.17 Ang.
PLAT234_ALERT_4_C Large Hirshfeld Difference C41      --C42      .       0.19 Ang.
PLAT234_ALERT_4_C Large Hirshfeld Difference O7       --C66      .       0.16 Ang.
PLAT234_ALERT_4_C Large Hirshfeld Difference N13      --C55      .       0.18 Ang.
PLAT234_ALERT_4_C Large Hirshfeld Difference N14      --C58      .       0.18 Ang.
PLAT234_ALERT_4_C Large Hirshfeld Difference N15      --C54      .       0.19 Ang.
PLAT234_ALERT_4_C Large Hirshfeld Difference N16      --C57      .       0.23 Ang.
PLAT234_ALERT_4_C Large Hirshfeld Difference N16      --C59      .       0.21 Ang.
PLAT234_ALERT_4_C Large Hirshfeld Difference C47      --C48      .       0.19 Ang.
PLAT234_ALERT_4_C Large Hirshfeld Difference C49      --C50      .       0.17 Ang.
PLAT234_ALERT_4_C Large Hirshfeld Difference C55      --C56      .       0.18 Ang.
PLAT234_ALERT_4_C Large Hirshfeld Difference C58      --C63      .       0.20 Ang.
PLAT234_ALERT_4_C Large Hirshfeld Difference C62      --C63      .       0.18 Ang.
PLAT234_ALERT_4_C Large Hirshfeld Difference C69      --C70      .       0.23 Ang.
PLAT241_ALERT_2_C High   'MainMol' Ueq as Compared to Neighbors of         N7 Check
PLAT241_ALERT_2_C High   'MainMol' Ueq as Compared to Neighbors of        C36 Check
PLAT241_ALERT_2_C High   'MainMol' Ueq as Compared to Neighbors of        C41 Check
PLAT241_ALERT_2_C High   'MainMol' Ueq as Compared to Neighbors of        C42 Check
PLAT241_ALERT_2_C High   'MainMol' Ueq as Compared to Neighbors of        N16 Check
PLAT241_ALERT_2_C High   'MainMol' Ueq as Compared to Neighbors of        C69 Check
PLAT241_ALERT_2_C High   'MainMol' Ueq as Compared to Neighbors of        C78 Check
PLAT241_ALERT_2_C High   'MainMol' Ueq as Compared to Neighbors of        C83 Check
```

```
PLAT242_ALERT_2_C Low    'MainMol' Ueq as Compared to Neighbors of        C7 Check
PLAT242_ALERT_2_C Low    'MainMol' Ueq as Compared to Neighbors of       C16 Check
PLAT242_ALERT_2_C Low    'MainMol' Ueq as Compared to Neighbors of       C54 Check
PLAT242_ALERT_2_C Low    'MainMol' Ueq as Compared to Neighbors of       C55 Check
PLAT242_ALERT_2_C Low    'MainMol' Ueq as Compared to Neighbors of       C58 Check
PLAT369_ALERT_2_C Long   C(sp2)-C(sp2) Bond  C13      - C21      .       1.55 Ang.
PLAT369_ALERT_2_C Long   C(sp2)-C(sp2) Bond  C55      - C56      .       1.54 Ang.
PLAT480_ALERT_4_C Long H...A H-Bond Reported H63      ..N10      .       2.68 Ang.
PLAT480_ALERT_4_C Long H...A H-Bond Reported H20      ..N1       .       2.67 Ang.
PLAT480_ALERT_4_C Long H...A H-Bond Reported H63      ..N10      .       2.68 Ang.
PLAT480_ALERT_4_C Long H...A H-Bond Reported H20      ..N1       .       2.67 Ang.
PLAT480_ALERT_4_C Long H...A H-Bond Reported H63      ..N10      .       2.68 Ang.
PLAT480_ALERT_4_C Long H...A H-Bond Reported H20      ..N1       .       2.67 Ang.
PLAT480_ALERT_4_C Long H...A H-Bond Reported H63      ..N10      .       2.68 Ang.
PLAT480_ALERT_4_C Long H...A H-Bond Reported H20      ..N1       .       2.67 Ang.
PLAT480_ALERT_4_C Long H...A H-Bond Reported H63      ..N10      .       2.68 Ang.
PLAT480_ALERT_4_C Long H...A H-Bond Reported H20      ..N1       .       2.67 Ang.
PLAT480_ALERT_4_C Long H...A H-Bond Reported H63      ..N10      .       2.68 Ang.
PLAT480_ALERT_4_C Long H...A H-Bond Reported H20      ..N1       .       2.67 Ang.
PLAT480_ALERT_4_C Long H...A H-Bond Reported H63      ..N10      .       2.68 Ang.
PLAT480_ALERT_4_C Long H...A H-Bond Reported H20      ..N1       .       2.67 Ang.
PLAT480_ALERT_4_C Long H...A H-Bond Reported H63      ..N10      .       2.68 Ang.
PLAT480_ALERT_4_C Long H...A H-Bond Reported H20      ..N1       .       2.67 Ang.
PLAT480_ALERT_4_C Long H...A H-Bond Reported H63      ..N10      .       2.68 Ang.
PLAT480_ALERT_4_C Long H...A H-Bond Reported H20      ..N1       .       2.67 Ang.
PLAT480_ALERT_4_C Long H...A H-Bond Reported H63      ..N10      .       2.68 Ang.
PLAT480_ALERT_4_C Long H...A H-Bond Reported H20      ..N1       .       2.67 Ang.
PLAT480_ALERT_4_C Long H...A H-Bond Reported H63      ..N10      .       2.68 Ang.
PLAT480_ALERT_4_C Long H...A H-Bond Reported H20      ..N1       .       2.67 Ang.
PLAT480_ALERT_4_C Long H...A H-Bond Reported H63      ..N10      .       2.68 Ang.
PLAT480_ALERT_4_C Long H...A H-Bond Reported H20      ..N1       .       2.67 Ang.
PLAT480_ALERT_4_C Long H...A H-Bond Reported H63      ..N10      .       2.68 Ang.
PLAT480_ALERT_4_C Long H...A H-Bond Reported H20      ..N1       .       2.67 Ang.
PLAT480_ALERT_4_C Long H...A H-Bond Reported H20      ..N1       .       2.67 Ang.
PLAT480_ALERT_4_C Long H...A H-Bond Reported H63      ..N10      .       2.68 Ang.
PLAT480_ALERT_4_C Long H...A H-Bond Reported H20      ..N1       .       2.67 Ang.
PLAT480_ALERT_4_C Long H...A H-Bond Reported H63      ..N10      .       2.68 Ang.
PLAT480_ALERT_4_C Long H...A H-Bond Reported H20      ..N1       .       2.67 Ang.
PLAT480_ALERT_4_C Long H...A H-Bond Reported H63      ..N10      .       2.68 Ang.
PLAT480_ALERT_4_C Long H...A H-Bond Reported H20      ..N1       .       2.67 Ang.
PLAT480_ALERT_4_C Long H...A H-Bond Reported H63      ..N10      .       2.68 Ang.
PLAT480_ALERT_4_C Long H...A H-Bond Reported H4       ..N1       .       2.71 Ang.
PLAT480_ALERT_4_C Long H...A H-Bond Reported H20      ..N1       .       2.67 Ang.
PLAT480_ALERT_4_C Long H...A H-Bond Reported H63      ..N10      .       2.68 Ang.
PLAT480_ALERT_4_C Long H...A H-Bond Reported H20      ..N1       .       2.67 Ang.
PLAT480_ALERT_4_C Long H...A H-Bond Reported H63      ..N10      .       2.68 Ang.
PLAT906_ALERT_3_C Large K Value in the Analysis of Variance ......     9.835 Check
PLAT906_ALERT_3_C Large K Value in the Analysis of Variance ......     2.486 Check
PLAT911_ALERT_3_C Missing FCF Refl Between Thmin & STh/L=    0.592       230 Report
PLAT975_ALERT_2_C Check Calcd Resid. Dens.  0.69A   From O13            0.57 eA-3
PLAT978_ALERT_2_C Number C-C Bonds with Positive Residual Density.         0 Info
```

Alert level G

```
PLAT003_ALERT_2_G Number of Uiso or Uij Restrained non-H Atoms ...        19 Report
PLAT007_ALERT_5_G Number of Unrefined Donor-H Atoms .............          6 Report
PLAT045_ALERT_1_G Calculated and Reported Z Differ by a Factor ...      0.25 Check
PLAT066_ALERT_1_G Predicted and Reported Tmin&Tmax Range Identical         ? Check
PLAT072_ALERT_2_G SHELXL First  Parameter in WGHT  Unusually Large      0.14 Report
PLAT177_ALERT_4_G The CIF-Embedded .res File Contains DELU Records          2 Report
PLAT178_ALERT_4_G The CIF-Embedded .res File Contains SIMU Records          1 Report
```

```
PLAT186_ALERT_4_G The CIF-Embedded .res File Contains ISOR Records          1 Report
PLAT199_ALERT_1_G Reported _cell_measurement_temperature ..... (K)        293 Check
PLAT200_ALERT_1_G Reported   _diffrn_ambient_temperature ..... (K)        293 Check
PLAT300_ALERT_4_G Atom Site Occupancy of O13        Constrained at        0.5 Check
PLAT300_ALERT_4_G Atom Site Occupancy of H113       Constrained at        0.5 Check
PLAT300_ALERT_4_G Atom Site Occupancy of H213       Constrained at        0.5 Check
PLAT302_ALERT_4_G Anion/Solvent/Minor-Residue Disorder (Resd  3  )       100% Note
PLAT304_ALERT_4_G Non-Integer Number of Atoms in ...... Resd  3          1.50 Check
PLAT720_ALERT_4_G Number of Unusual/Non-Standard Labels ..........          4 Note
PLAT779_ALERT_4_G Suspect or Irrelevant (Bond) Angle in CIF .... #         75 Check
              H213 -O13  -H213    1.555   1.555   1.555              0.00 Deg.
PLAT794_ALERT_5_G Tentative Bond Valency for Fe1       (III)     .       3.23 Info
PLAT794_ALERT_5_G Tentative Bond Valency for Fe2       (III)     .       3.33 Info
PLAT794_ALERT_5_G Tentative Bond Valency for Mn1       (I)       .       0.85 Info
PLAT794_ALERT_5_G Tentative Bond Valency for Mn2       (I)       .       0.84 Info
PLAT860_ALERT_3_G Number of Least-Squares Restraints .............         39 Note
PLAT883_ALERT_1_G No Info/Value for _atom_sites_solution_primary .     Please Do !
PLAT910_ALERT_3_G Missing # of FCF Reflection(s) Below Theta(Min).          4 Note
PLAT933_ALERT_2_G Number of OMIT Records in Embedded .res File ...         68 Note
```

1 **ALERT level A** = Most likely a serious problem - resolve or explain
2 **ALERT level B** = A potentially serious problem, consider carefully
90 **ALERT level C** = Check. Ensure it is not caused by an omission or oversight
25 **ALERT level G** = General information/check it is not something unexpected

5 ALERT type 1 CIF construction/syntax error, inconsistent or missing data
25 ALERT type 2 Indicator that the structure model may be wrong or deficient
8 ALERT type 3 Indicator that the structure quality may be low
75 ALERT type 4 Improvement, methodology, query or suggestion
5 ALERT type 5 Informative message, check

It is advisable to attempt to resolve as many as possible of the alerts in all categories. Often the minor alerts point to easily fixed oversights, errors and omissions in your CIF or refinement strategy, so attention to these fine details can be worthwhile. In order to resolve some of the more serious problems it may be necessary to carry out additional measurements or structure refinements. However, the purpose of your study may justify the reported deviations and the more serious of these should normally be commented upon in the discussion or experimental section of a paper or in the "special_details" fields of the CIF. checkCIF was carefully designed to identify outliers and unusual parameters, but every test has its limitations and alerts that are not important in a particular case may appear. Conversely, the absence of alerts does not guarantee there are no aspects of the results needing attention. It is up to the individual to critically assess their own results and, if necessary, seek expert advice.

Publication of your CIF in IUCr journals

A basic structural check has been run on your CIF. These basic checks will be run on all CIFs submitted for publication in IUCr journals (*Acta Crystallographica*, *Journal of Applied Crystallography*, *Journal of Synchrotron Radiation*); however, if you intend to submit to *Acta Crystallographica Section C* or *E* or *IUCrData*, you should make sure that full publication checks are run on the final version of your CIF prior to submission.

Publication of your CIF in other journals

Please refer to the *Notes for Authors* of the relevant journal for any special instructions relating to CIF submission.

PLATON version of 03/05/2019; check.def file version of 29/04/2019

Datablock 3 - ellipsoid plot

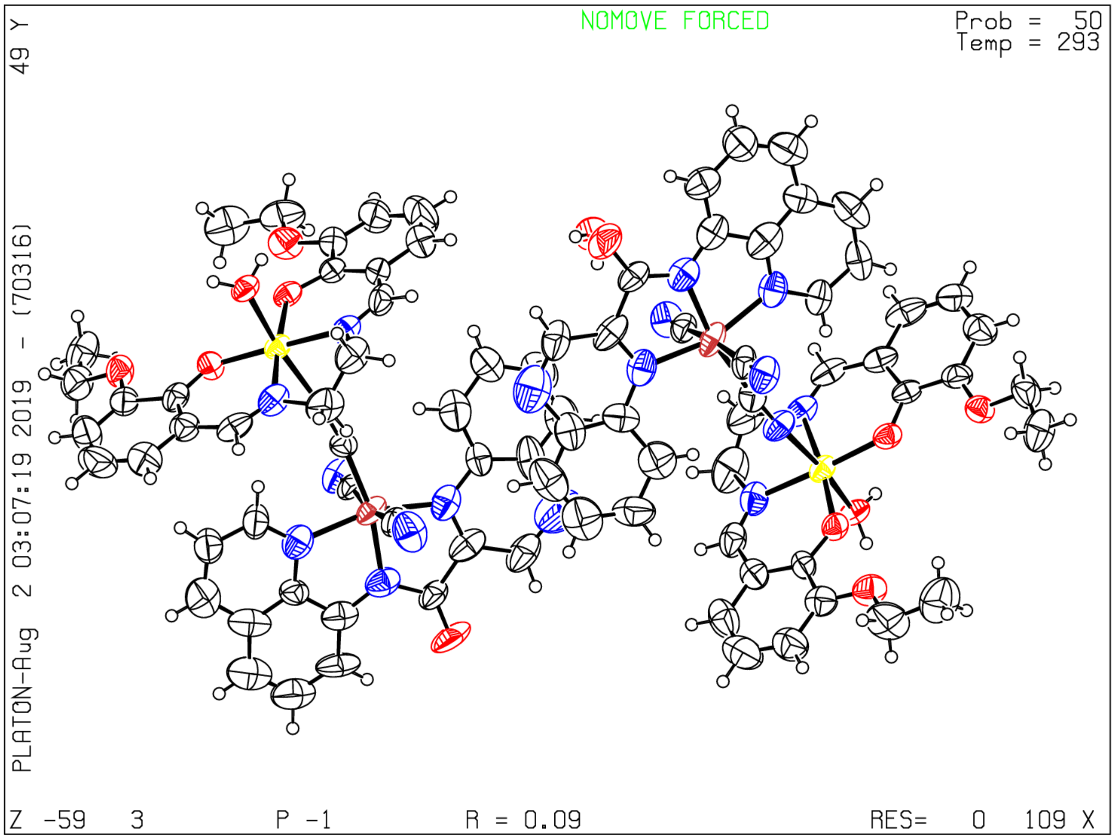

Supplement: Supplementary file 1 [file checkcif 3.pdf › checkcif 3.pdf]
