# Supplementary material for: Various Structural Types of Cyanide-Bridged FeIII–MnIII Bimetallic Coordination Polymers (CPs) and Polynuclear Clusters Based-on A New mer-Tricyanoiron(III)Building Block: Synthesis, Crystal Structures, and Magnetic Properties
Source: Polymers (Basel). 2019 Sep 27;11(10):1585. doi: 10.3390/polym11101585 (PMC6835830; doi:10.3390/polym11101585)

checkCIF/PLATON report

Structure factors have been supplied for datablock(s) test

THIS REPORT IS FOR GUIDANCE ONLY. IF USED AS PART OF A REVIEW PROCEDURE FOR PUBLICATION, IT SHOULD NOT REPLACE THE EXPERTISE OF AN EXPERIENCED CRYSTALLOGRAPHIC REFEREE.

No syntax errors found. CIF dictionary Interpreting this report

Datablock: test

```
Bond precision:  C-C = 0.0246 A          Wavelength=0.71073

Cell:             a=8.886(3)     b=19.845(6)         c=19.662(5)
                  alpha=90       beta=99.847(6)      gamma=90
Temperature:      293 K

                Calculated                Reported
Volume          3416.2(18)                3416.0(17)
Space group     P 21/n                    P 21/n
Hall group      -P 2yn                    -P 2yn
Moiety formula  C33 H29 Fe Mn N9 O3       ?
Sum formula     C33 H29 Fe Mn N9 O3       C33 H29 Fe Mn N9 O3
Mr              710.44                    710.44
Dx,g cm-3       1.381                     1.381
Z               4                         4
Mu (mm-1)       0.841                     0.841
F000            1460.0                    1460.0
F000'           1463.15
h,k,lmax        9,22,21                   9,22,21
Nref            4950                      4907
Tmin,Tmax       0.896,0.935               0.898,0.936
Tmin'           0.896

Correction method= # Reported T Limits: Tmin=0.898 Tmax=0.936
AbsCorr = MULTI-SCAN

Data completeness= 0.991          Theta(max)= 23.331

R(reflections)= 0.0960( 3285)     wR2(reflections)= 0.2797( 4907)

S = 1.015                  Npar= 428
```

The following ALERTS were generated. Each ALERT has the format
test-name_ALERT_alert-type_alert-level.
Click on the hyperlinks for more details of the test.

Alert level B

```
THETM01_ALERT_3_B  The value of sine(theta_max)/wavelength is less than 0.575
            Calculated sin(theta_max)/wavelength =    0.5572
```

Author Response: The crystal is very small and fragile, making the diffraction very weak, especially in high angle range.

```
PLAT341_ALERT_3_B Low Bond Precision on  C-C Bonds ...............    0.02462 Ang.
PLAT601_ALERT_2_B Structure Contains Solvent Accessible VOIDS of .        149 Ang**3
```

Alert level C

```
PLAT084_ALERT_3_C High wR2 Value (i.e. > 0.25) ...................       0.28 Report
PLAT220_ALERT_2_C Non-Solvent  Resd 1  C   Ueq(max)/Ueq(min) Range        3.1 Ratio
PLAT232_ALERT_2_C Hirshfeld Test Diff (M-X)  Fe1      --N6       .        5.4 s.u.
PLAT234_ALERT_4_C Large Hirshfeld Difference Fe1      --N4       .       0.16 Ang.
PLAT234_ALERT_4_C Large Hirshfeld Difference O2       --C29      .       0.18 Ang.
PLAT234_ALERT_4_C Large Hirshfeld Difference N7       --C16      .       0.22 Ang.
PLAT234_ALERT_4_C Large Hirshfeld Difference N8       --C31      .       0.16 Ang.
PLAT234_ALERT_4_C Large Hirshfeld Difference C10      --C11      .       0.18 Ang.
PLAT241_ALERT_2_C High   'MainMol' Ueq as Compared to Neighbors of         N7 Check
PLAT241_ALERT_2_C High   'MainMol' Ueq as Compared to Neighbors of         C5 Check
PLAT241_ALERT_2_C High   'MainMol' Ueq as Compared to Neighbors of        C17 Check
PLAT241_ALERT_2_C High   'MainMol' Ueq as Compared to Neighbors of        C22 Check
PLAT241_ALERT_2_C High   'MainMol' Ueq as Compared to Neighbors of        C25 Check
PLAT241_ALERT_2_C High   'MainMol' Ueq as Compared to Neighbors of        C30 Check
PLAT242_ALERT_2_C Low    'MainMol' Ueq as Compared to Neighbors of         N8 Check
PLAT242_ALERT_2_C Low    'MainMol' Ueq as Compared to Neighbors of         C7 Check
PLAT242_ALERT_2_C Low    'MainMol' Ueq as Compared to Neighbors of        C16 Check
PLAT242_ALERT_2_C Low    'MainMol' Ueq as Compared to Neighbors of        C24 Check
PLAT242_ALERT_2_C Low    'MainMol' Ueq as Compared to Neighbors of        C26 Check
PLAT242_ALERT_2_C Low    'MainMol' Ueq as Compared to Neighbors of        C31 Check
PLAT411_ALERT_2_C Short Inter H...H Contact  H17      ..H17      .       2.13 Ang.
                                                2-x,1-y,2-z  =      3_767 Check
PLAT911_ALERT_3_C Missing FCF Refl Between Thmin & STh/L=    0.557         43 Report
PLAT978_ALERT_2_C Number C-C Bonds with Positive Residual Density.          0 Info
```

Alert level G

```
PLAT003_ALERT_2_G Number of Uiso or Uij Restrained non-H Atoms ...         20 Report
PLAT004_ALERT_5_G Polymeric Structure Found with Maximum Dimension          1 Info
PLAT066_ALERT_1_G Predicted and Reported Tmin&Tmax Range Identical          ? Check
PLAT072_ALERT_2_G SHELXL First  Parameter in WGHT  Unusually Large       0.12 Report
PLAT083_ALERT_2_G SHELXL Second Parameter in WGHT  Unusually Large      12.17 Why ?
PLAT177_ALERT_4_G The CIF-Embedded .res File Contains DELU Records          2 Report
PLAT178_ALERT_4_G The CIF-Embedded .res File Contains SIMU Records          1 Report
PLAT186_ALERT_4_G The CIF-Embedded .res File Contains ISOR Records          1 Report
PLAT199_ALERT_1_G Reported _cell_measurement_temperature ..... (K)        293 Check
PLAT200_ALERT_1_G Reported   _diffrn_ambient_temperature ..... (K)        293 Check
PLAT335_ALERT_2_G Check Large C6 Ring C-C Range C7       -C12            0.21 Ang.
PLAT335_ALERT_2_G Check Large C6 Ring C-C Range C15      -C20            0.23 Ang.
PLAT794_ALERT_5_G Tentative Bond Valency for Fe1       (III)     .       3.31 Info
PLAT794_ALERT_5_G Tentative Bond Valency for Mn1       (I)       .       0.83 Info
PLAT860_ALERT_3_G Number of Least-Squares Restraints .............         57 Note
```

```
PLAT883_ALERT_1_G No Info/Value for _atom_sites_solution_primary .     Please Do !
PLAT910_ALERT_3_G Missing # of FCF Reflection(s) Below Theta(Min).          1 Note
PLAT933_ALERT_2_G Number of OMIT Records in Embedded .res File ...         23 Note
```

```
   0 ALERT level A = Most likely a serious problem - resolve or explain
   3 ALERT level B = A potentially serious problem, consider carefully
  23 ALERT level C = Check. Ensure it is not caused by an omission or oversight
  18 ALERT level G = General information/check it is not something unexpected

   4 ALERT type 1 CIF construction/syntax error, inconsistent or missing data
  23 ALERT type 2 Indicator that the structure model may be wrong or deficient
   6 ALERT type 3 Indicator that the structure quality may be low
   8 ALERT type 4 Improvement, methodology, query or suggestion
   3 ALERT type 5 Informative message, check
```

It is advisable to attempt to resolve as many as possible of the alerts in all categories. Often the minor alerts point to easily fixed oversights, errors and omissions in your CIF or refinement strategy, so attention to these fine details can be worthwhile. In order to resolve some of the more serious problems it may be necessary to carry out additional measurements or structure refinements. However, the purpose of your study may justify the reported deviations and the more serious of these should normally be commented upon in the discussion or experimental section of a paper or in the "special_details" fields of the CIF. checkCIF was carefully designed to identify outliers and unusual parameters, but every test has its limitations and alerts that are not important in a particular case may appear. Conversely, the absence of alerts does not guarantee there are no aspects of the results needing attention. It is up to the individual to critically assess their own results and, if necessary, seek expert advice.

Publication of your CIF in IUCr journals

A basic structural check has been run on your CIF. These basic checks will be run on all CIFs submitted for publication in IUCr journals (*Acta Crystallographica*, *Journal of Applied Crystallography*, *Journal of Synchrotron Radiation*); however, if you intend to submit to *Acta Crystallographica Section C* or *E* or *IUCrData*, you should make sure that full publication checks are run on the final version of your CIF prior to submission.

Publication of your CIF in other journals

Please refer to the *Notes for Authors* of the relevant journal for any special instructions relating to CIF submission.

PLATON version of 03/05/2019; check.def file version of 29/04/2019

Datablock test - ellipsoid plot

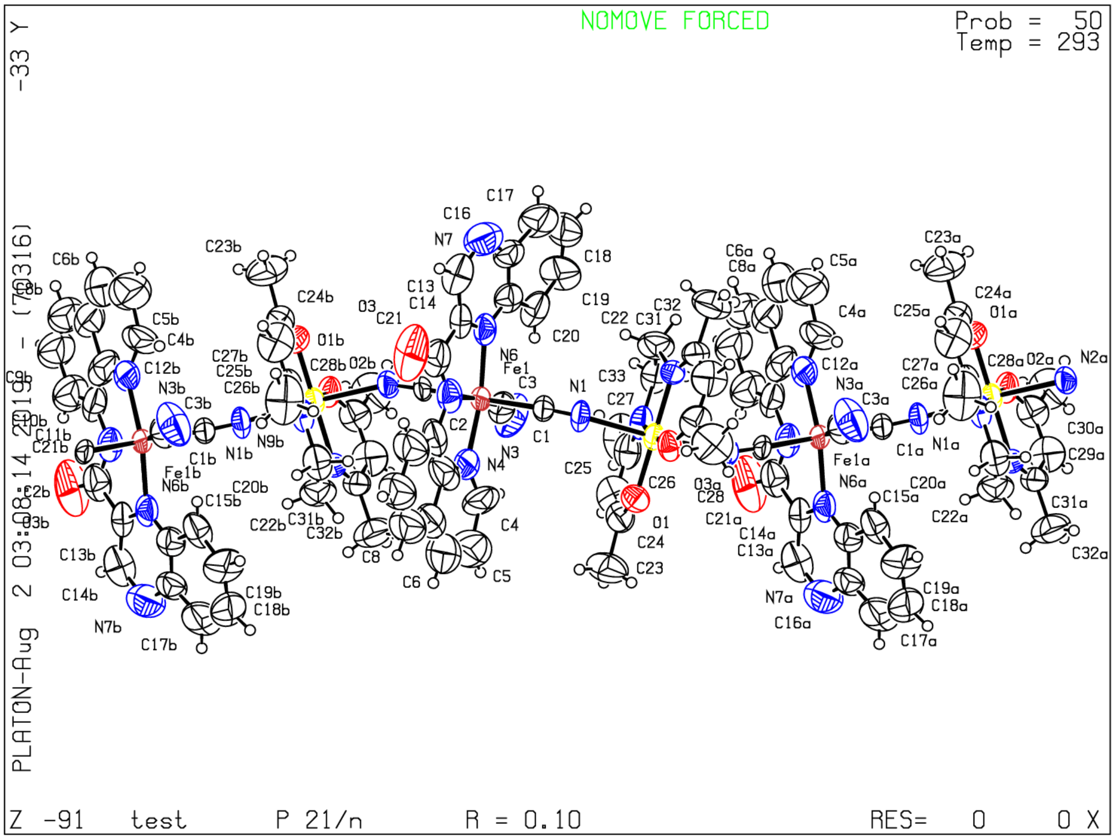

Supplement: Supplementary file 1 [file checkcif 4.pdf › checkcif 4.pdf]
